# Cardiac inflammatory CD11b/c cells exert a protective role in hypertrophied cardiomyocyte by promoting TNFR_2_- and Orai3- dependent signaling

**DOI:** 10.1038/s41598-019-42452-y

**Published:** 2019-04-15

**Authors:** Mathilde Keck, Mathilde Flamant, Nathalie Mougenot, Sophie Favier, Fabrice Atassi, Camille Barbier, Sophie Nadaud, Anne-Marie Lompré, Jean-Sébastien Hulot, Catherine Pavoine

**Affiliations:** 1 Sorbonne Universités, UPMC Univ Paris 06, INSERM, Institute of Cardiometabolism and Nutrition (ICAN), Team 3, F-75013 Paris, France; 2 UMS28, plateforme PECMV, F-75013 Paris, France

## Abstract

Early adaptive cardiac hypertrophy (EACH) is initially a compensatory process to optimize pump function. We reported the emergence of Orai3 activity during EACH. This study aimed to characterize how inflammation regulates store-independent activation of Orai3-calcium influx and to evaluate the functional role of this influx. Isoproterenol infusion or abdominal aortic banding triggered EACH. TNFα or conditioned medium from cardiac CD11b/c cells activated either *in vivo* [isolated from rats displaying EACH], or *in vitro* [isolated from normal rats and activated with lipopolysaccharide], were added to adult cardiomyocytes before measuring calcium entry, cell hypertrophy and cell injury. Using intramyocardial injection of siRNA, Orai3 was *in vivo* knockdown during EACH to evaluate its protective activity in heart failure. Inflammatory CD11b/c cells trigger a store-independent calcium influx in hypertrophied cardiomyocytes, that is mimicked by TNFα. Pharmacological or molecular (siRNA) approaches demonstrate that this calcium influx, depends on TNFR_2_, is Orai3-driven, and elicits cardiomyocyte hypertrophy and resistance to oxidative stress. Neutralization of Orai3 inhibits protective GSK3β phosphorylation, impairs EACH and accelerates heart failure. Orai3 exerts a pathophysiological protective impact in EACH promoting hypertrophy and resistance to oxidative stress. We highlight inflammation arising from CD11b/c cells as a potential trigger of TNFR_2_- and Orai3-dependent signaling pathways.

## Introduction

Cardiac hypertrophy (CH) is initially a compensatory process to optimize cardiac pump function^1^. However, CH is progressively associated with structural changes that become pathogenic, with cardiomyocyte death, induction of exacerbated inflammatory responses and interstitial fibrosis. These harmful changes ultimately lead to transition to heart failure (HF). Activation of the sympathetic nervous system plays a determinant role in the induction of early adaptive CH (EACH) and further progression to pathological remodeling^2,3^. HF is a major health issue^4^ and a better understanding of cellular mechanisms elicited during EACH is needed to prevent the progression to HF or favor recovery^5,6^.

Altered myocardial calcium (Ca^2+^) cycling is a hallmark of HF underlying perturbation in excitation-contraction coupling^7^. Voltage-gated ion channels, the sarcoplasmic reticulum Ca^2+^ ATPase, the Na^+^-Ca^2+^ exchanger, the ryanodine receptor and t-tubule structure became promising targets for therapeutical intervention. In addition, Ca^2+^ handling remodeling also drives hypertrophic and apoptotic responses. In this context, TRPCs (canonical transient receptor potential channels)-, STIM1 (stromal interaction molecule 1)-, and Orai1-dependent Ca^2+^ entry are instrumental for pathological left ventricular hypertrophy development^8–13^. As described in the non-excitable cells, TRPCs, STIM1 and Orai1 molecules drive store-operated Ca^2+^ entry^10,11,13–15^. An alternative Ca^2+^ entry pathway, independent of store-depletion, involves the key participation of the Orai3 molecule^14–20^. Orai3-driven store-independent Ca^2+^ entry relies on initial arachidonic acid (AA) production, and is selectively activated by AA itself (ARC channels) or its leukotriene C4 (LTC4) metabolite (LRC channels), in all cell types examined to date. Knowledge regarding Orai3 contribution to cardiac remodeling remains scarce. We recently demonstrated the emergence of an Orai3-dependent pathway that drives an AA-dependent Ca^2+^ influx in hypertrophied cardiomyocytes from rats subjected to abdominal aortic banding^12^. This study documented the essential role of constitutive Orai3-dependent activity to initiate and maintain early adaptive hypertrophy in response to pressure overload. But pathophysiological triggers and mechanisms leading to Orai3 activation during EACH remained unknown, as well as its direct impact on cardiomyocytes and its functional relevance in HF.

Cardiac remodeling is a complex inflammatory syndrome^5^, and beneficial or detrimental role of inflammatory signaling during EACH is not fully understood. Growing evidence indicates that inflammatory responses emerging in EACH and HF are different, displaying divergent cytokine profiling^21^. The pro-inflammatory cytokine TNFα is upregulated in CH and HF. In the 1990’s, the “cytokine hypothesis” argued for the detrimental contribution of an excessive production of TNFα to the pathogenesis of HF^22^, via binding to the TNFR_1_ receptor subtype, suggesting that TNFα neutralization would be beneficial. Surprisingly, large clinical trials failed to demonstrate a benefit of anti-TNFα strategies^23,24^. There is now evidence that TNFα can also improve remodeling and hypertrophy and alleviate inflammation and fibrosis upon binding to the TNFR_2_ receptor subtype or regulation of TNFR_1_ signaling, in cardiomyocytes, or indirectly after induction of GM-CSF secretion by endothelial renal cells, or influencing cardiac immune cell phenotypes^2,25–29^. In this context, we have previously shown that AA mediates dual effect of TNFα on Ca^2+^ transients and contraction of adult rat myocytes^30^ and identified TNFR_2_-dependent activation of the cytosolic phospholipase A_2_ (cPLA_2_) activity as a pathway leading to AA production and conferring resistance of adult cardiomyocytes to H_2_O_2_^26^. Recent studies suggested the potential adaptive role of TNFα in early cardiac remodeling showing that myocardial gene expression of TNFα is significantly higher in patients with well compensated aortic stenosis than in patients with decompensated stenosis^31^ and the association of circulating TNFα with concentric left ventricular remodeling^32^. The present study aimed to investigate the potential regulation of the AA-dependent Orai3 influx by TNFα in early adaptive cardiac remodeling, identify the potential cellular source of such an inflammatory signal, evaluate the impact of TNFα-induced Orai3 regulation on cardiomyocyte hypertrophy and resistance to H_2_O_2,_ and assess the functional relevance of Orai3 activity in HF.

Our study points out a novel TNFR_2_-dependent signaling pathway in cardiomyocytes that triggers Orai3-driven Ca^2+^ influx enhancing hypertrophy and promoting an increased resistance to oxidative stress. Cardiac CD11b/c cells arise as a potential source of this protective inflammatory stimulus. Neutralization of Orai3 during EACH fosters evolution towards HF.

## Results

### TNFα triggers activation of Orai3-Ca^2+^ influx in hypertrophied cardiomyocytes

**Figure 1:**
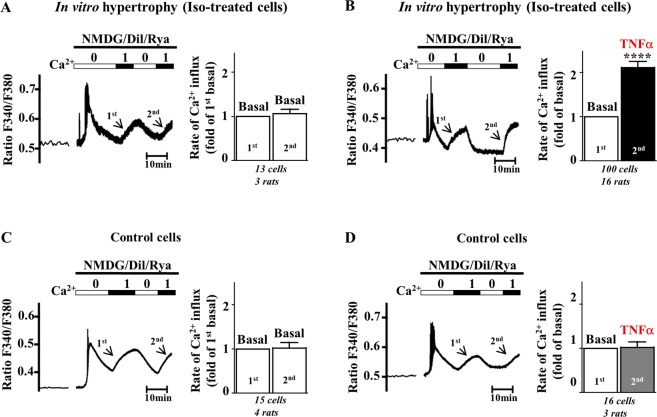
TNFα activates a store- and voltage-independent Ca^2+^ influx in hypertrophied but not in control cardiomyocytes. Representative recordings of Fura_2_ fluorescence ratio (F340/F380) in iso-treated hypertrophied (**A**,**B**) or control (**C**,**D**) cardiomyocytes subjected to 2 successive measurements of the rate of voltage- and store-independent Ca^2+^ entry and quantification of rates of Ca^2+^ entry. Two successive applications of the same 1 mM Ca^2+^ basal perfusion medium gave rise to similar rates of Ca^2+^ entry, both in hypertrophied (**A**) and normal cells (**C**). Addition of TNFα to the second 1 mM Ca^2+^ perfusion medium enhanced the rate of Ca^2+^ entry (2^nd^ slope as compared to 1^st^ slope) in hypertrophied cardiomyocytes (**B**) but not in normal cardiomyocytes (**D**). Number of cells analyzed and number of cell isolations (rats) as indicated. Mean ± SEM of cells, Wilcoxon matched-paired tests to examine if the mean of the 2^nd^ rate differs from the 1^st^ one, arbitrarily set as 1, ****p < 0.0001.

To investigate the regulation of the Orai3-Ca^2+^ influx by TNFα, we first used adult cardiomyocytes isolated from normal rats and incubated or not with isoproterenol (iso) (100 nM overnight) to elicit *in vitro* hypertrophy, as demonstrated by an increased cell area (2256 ± 37 *vs*. 2541 ± 53 µm^2^, n = 308 control *vs*. 313 Iso cells, *p* < 0.0001, Mann Whitney U test). We performed Ca^2+^-imaging experiments in Fura_2_-loaded cardiomyocytes to directly evaluate the impact of inflammation on Orai-dependent Ca^2+^ influx. After electrical stimulation as a quality test, cells were placed in a medium appropriate for the measurement of voltage- and store-independent Ca^2+^ influx containing diltiazem and ryanodine where Na^+^ was replaced by the large organic ion N-methyl-D-glucamine, as previously reported^12^ (Fig. S1). After equilibration in the absence of Ca^2+^, 1 mmol/L Ca^2+^ was added into the extracellular medium and the resultant initial increase in Fura_2_ fluorescence (1^st^ slope) was taken as an index of initial rate of Ca^2+^ influx. This protocol was routinely applied a second time (2^nd^ slope) to allow paired comparison, either between two identical perfusion conditions and to ascertain reproducibility of measurements (Fig. 1A,C), or between two different perfusion conditions (Fig. 1B,D). Two successive applications of the same “basal” medium gave rise to similar rates of Ca^2+^ influx, either in control or hypertrophied cardiomyocytes (Fig. 1A,C). In contrast, addition of TNFα to the second incubation medium induced a significant increase in the rate of Ca^2+^ influx (2^nd^ slope) as compared to basal (1^st^ slope). Importantly, this effect was selectively detected in hypertrophied cardiomyocytes (Fig. 1B) but not in control ones (Fig. 1D). These data show that TNFα selectively induces Ca^2+^ influx in hypertrophied cardiomyocytes.

**Figure 2:**
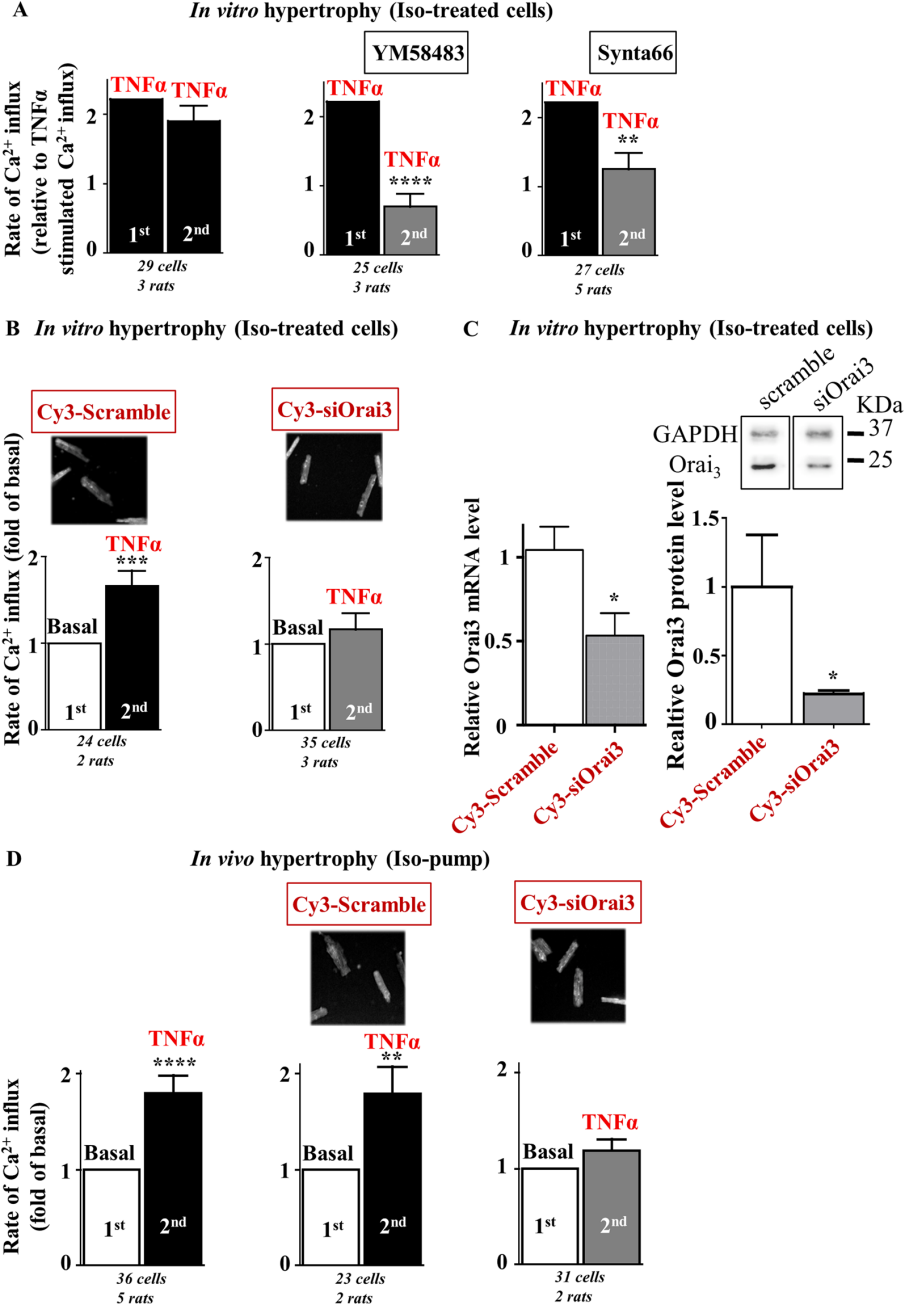
TNFα activates a store- and voltage-independent Ca^2+^ influx in hypertrophied cardiomyocytes further identified as Orai3 dependent. Hypertrophied cardiomyocytes were loaded with Fura_2_ before measurement of voltage- and store-independent Ca^2+^ influx. (**A**) Two successive applications of the same TNFα perfusion medium on iso-treated hypertrophied cardiomyocytes gave rise to similar rates of Ca^2+^ entry. The first TNFα-induced Ca^2+^ influx was arbitrarily set to 2.2 to allow comparisons with the control conditions (see Fig. 1B). Preincubation with Orai inhibitors, YM58483 or Synta66, prior the second application of TNFα perfusion medium blunted activation of Ca^2+^ entry by TNFα. (**B**) Hypertrophied cardiomyocytes isolated from hearts injected with Cy3-tagged scramble or Orai3 siRNAs three days before isolation and iso-treatment. Typical images show Cy3-fluorescence in cardiomyocytes. TNFα activates Ca^2+^ influx in scramble siRNA-transfected cells but not in siOrai3-transfected cardiomyocytes. Number of cells analyzed and number of cell isolations (rats) as indicated, mean ± SEM of cells, Wilcoxon matched-paired tests to examine if the mean of the 2^nd^ rate differs from the 1^st^ one, arbitrarily set as 2.2 (**A**) or 1 (**B**), **p < 0.01, ***p < 0.001, ****p < 0.0001. (**C**) Efficient knockdown of Orai3 mRNA and protein in cardiomyocytes isolated from Wistar rats at day 3 following injection with Cy3-tagged scramble or Orai3 siRNAs. Histograms representing relative transcript levels normalized to the RPL32 mRNA or relative protein levels normalized to Glyceraldehyde 3-phosphate dehydrogenase (GAPDH) protein. Mean ± SEM of cardiomyocytes from 2–6 rats/group, Mann-Whitney U test, *p < 0.05. Full length blots were included in SI. (**D**) Hypertrophied cardiomyocytes isolated from rats after fourteen days of chronic iso-infusion injected with Cy3-tagged scramble or Orai3 siRNAs three days before isolation. TNFα activates Ca^2+^ influx in hypertrophied cardiomyocytes isolated from hearts injected or not with Cy3-tagged scramble siRNA, but not in hypertrophied cardiomyocytes isolated from hearts injected with Cy3-tagged Orai3 siRNA, three days before isolation. Number of cells analyzed and number of cell isolations (rats) as indicated, mean ± SEM of cells, Wilcoxon matched-paired tests to examine if the mean of the 2^nd^ rate differs from the 1^st^ one, arbitrarily set as 1, **p < 0.01, ****p < 0.0001.

We then asked whether Orai3 drives this TNFα-activated store- and voltage-independent Ca^2+^ influx. In *in vitro* iso-treated hypertrophied cells, two successive applications of identical TNFα containing medium gave rise to similar rates of Ca^2+^ influx (Fig. 2A left). However, preincubation for 10 min with Orai pharmacological inhibitors, YM58483 or Synta66, reduced the rate of Ca^2+^ influx observed in response to a second challenge with TNFα, to a value similar to basal (Fig. 2A middle and right). Moreover, *in vivo* molecular knockdown of Orai3, via intramyocardial injection with Cy3-tagged Orai3 siRNA, also blunted the TNFα effect on Ca^2+^ influx that was still observed in cells isolated from scramble siRNA-injected hearts (Fig. 2B). Orai3 neutralization was performed as previously reported^12^ and demonstrated by quantitative RT-PCR and Western-blot (Fig. 2C) and by detection of Cy3 positive cells (Fig. 2B). Of note, knockdown of Orai3 by siRNA injection in normal rats did not modify the cardiomyocyte size (Table S1). In contrast, both siScramble and siOrai3 cardiomyocytes had a tendency to be bigger after *in vitro* post-treatment with iso and displayed similar sizes. This suggested that the *in vitro* iso-hypertrophic response was not altered in siOrai3 cardiomyocyte, in contrast to TNFα/Orai3 signaling.

Complementary experiments were performed in hearts from rats subjected to chronic iso-infusion for 14 days (1.5 mg/kg/day) to elicit *in vivo* hypertrophy (Table 1). Iso-induced EACH remodeling was confirmed by an increase in end-diastolic and end-systolic interventricular septum (IVSd and IVSs), posterior wall thicknesses (PWd and PWs) and concentric hypertrophy (h/r, diastolic wall thickness to radius ratio) in iso-treated rats, relative to control rats, associated with an increased heart rate (HR) and a better cardiac function assessed by fractional shortening measurements (FS).

**Table 1:** echocardiography parameters at day 14 in control or iso-infused rats.

	d14 control rats (n = 7)	d14 iso-treated rats (n = 21)	Mann-Whitney U test
HR (bpm)	390 ± 7	491 ± 4	p < 0.0001
IVSd (mm)	1.47 ± 0.04	1.93 ± 0.03	p < 0.0001
LVd (mm)	7.67 ± 0.16	7.37 ± 0.07	ns
PWd (mm)	1.89 ± 0.02	2.3 ± 0.05	p < 0.0001
IVSs (mm)	2.44 ± 0.08	3.11 ± 0.07	p < 0.0001
LVs (mm)	4.31 ± 0.07	3.51 ± 0.0001	p < 0.0001
PWs (mm)	2.7 ± 0.06	3.18 ± 0.06	p < 0.0001
h/r	0.44 ± 0.01	0.57 ± 0.01	p < 0.0001
EF (%)	79.98 ± 0.45	87.2 ± 0.65	p < 0.0001
FS (%)	43.6 ± 0.5	51.3 ± 1.1	p < 0.001

HR, heart rate; IVSd, end-diastolic interventricular septum thickness; LVd, end-diastolic left ventricular diameter; PWd, end-diastolic posterior wall thickness; IVSs, end-systolic interventricular septum thickness; LVs, end-systolic left ventricular diameter; PWs, end-systolic posterior wall thickness; h/r, diastolic wall thickness to radius ratio; EF, ejection fraction; FS, fractional shortening.

Iso-induced hypertrophy was also attested by an increased heart weight (HW) to body weight (BW) ratio (0.84 ± 0.12 *vs*. 0.62 ± 0.11, n = 8 Iso *vs*. 8 control rats, *p* < 0.01, Mann Whitney U test) and cell area (3128 ± 63 *vs*. 2538 ± 54 µm^2^, n = 214 Iso *vs*. 223 control cells, *p* < 0.0001, Mann Whitney U test).

Ca^2+^-imaging experiments confirmed that TNFα also activated Orai3-dependent Ca^2+^ influx in *in vivo* hypertrophied cardiomyocytes isolated from rats implanted with iso-pump and injected or not with siRNAs three days before cardiomyocyte isolation (Fig. 2D). Of note, efficient Orai3 knockdown in *in vivo* hypertrophied cardiomyocytes was attested by a 40.0 ± 14.6% decrease in Orai3 mRNA expression as compared to cardiomyocytes isolated from scramble siRNA injected rats (*p* < 0.05, Mann-Whithney U test, n = 3 Orai3 and n = 6 Scramble siRNA injected rats). Moreover, *in vivo* reduction in Orai3 expression in iso-infused rats decreased the mean cardiomyocyte area (3384 ± 81 *vs*. 4311 ± 135 µm^2^, n = 116 *vs*. 120 cells from Orai3 *vs*. Scramble siRNA injected rat hearts at day 3 post injection, *p* < 0.0001, Mann Whitney U test). These results highlighted the role of Orai3-dependent Ca^2+^ influx as a target of TNFα in *in vitro* and *in vivo* iso-hypertrophied cardiomyocytes.

Overall, these experiments identified TNFα as an activator of the Orai3-dependent Ca^2+^ influx in hypertrophied cardiomyocytes.

### Activation of Orai3-Ca^2+^ influx by TNFα relies on binding to TNFR_2_, stimulation of cPLA_2_ and potential production of AA metabolites via the lipoxygenase pathway

**Figure 3:**
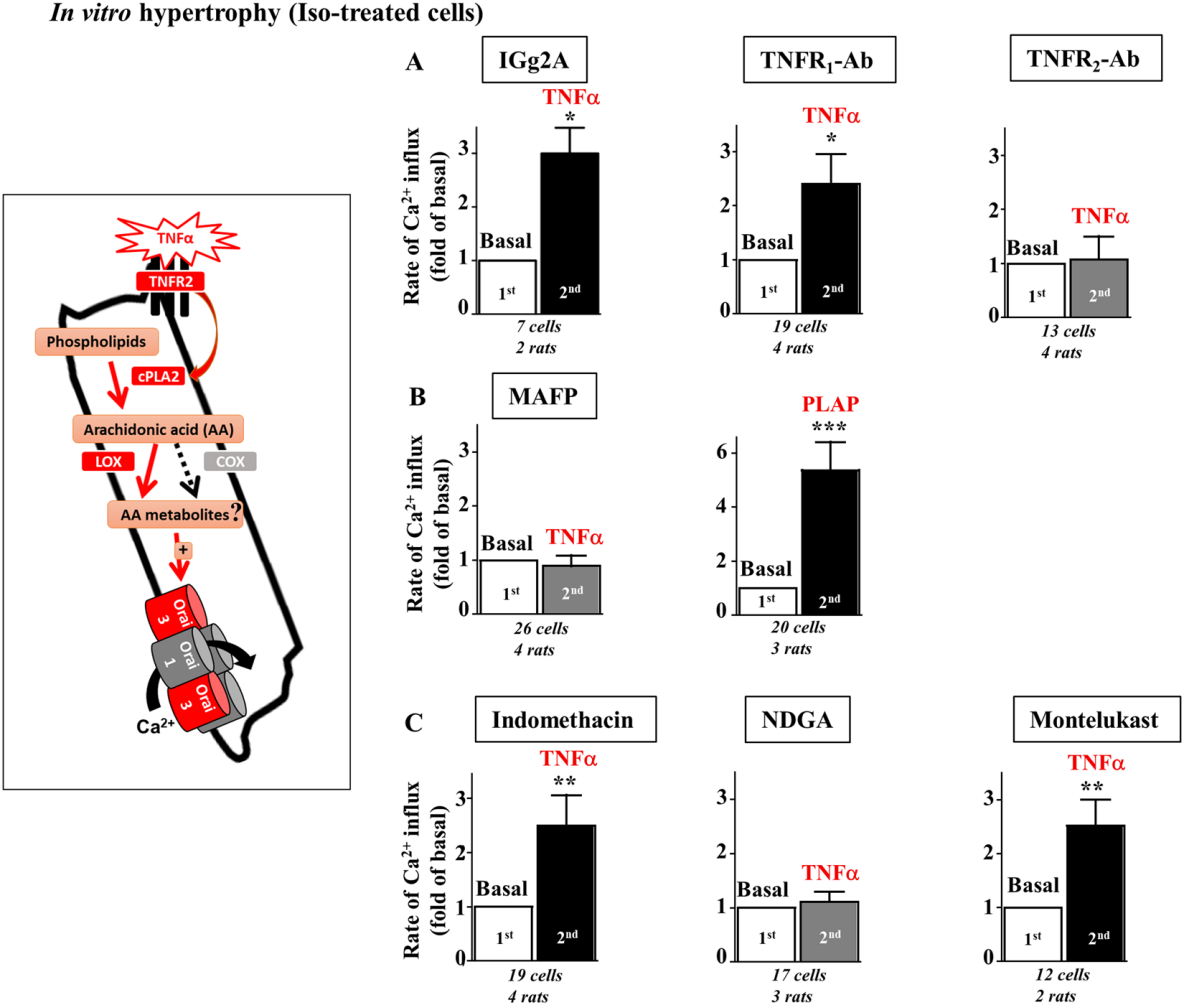
Activation by TNFα relies on binding to TNFR_2_, stimulation of cPLA_2_ and potential production of AA metabolites via the lipoxygenase pathway. (**A**–**C**) Iso-treated hypertrophied cardiomyocytes were loaded with Fura_2_ before measurement of voltage- and store-independent Ca^2+^ influx. (**A**) Rates of Ca^2+^ entry in response to TNFα after a 1 hour preincubation with control IGg2A, neutralizing TNFR_1_-Ab or TNFR_2_-Ab. (**B**) TNFα effect is sensitive to the cPLA_2_ inhibitor, MAFP, but mimicked by cPLA_2_ activating peptide PLAP. (**C**) Activation of Ca^2+^ entry by TNFα is blunted by preincubation with NDGA (lipoxygenase inhibitor), but unaffected by indomethacin (cyclooxygenase inhibitor) or montelukast (leukotriene receptor antagonist) pretreatments. Number of cells analyzed and number of cell isolations (rats) as indicated. Mean ± SEM of cells, Wilcoxon matched-paired tests to examine if the mean of the 2^nd^ rate differs from the 1^st^ one, arbitrarily set as 1, *p < 0.05, **p < 0.01, ***p < 0.001.

Next we aimed to evaluate the role of TNFα receptors 1 and 2 and of the cPLA_2_ pathways in TNFα signaling. In *in vitro* iso-treated hypertrophied cells, stimulation of Ca^2+^ influx by TNFα was unaffected by the preincubation for 1 hour either with control IGg2A or anti-TNFR_1_-antibodies (Ab) but was impaired in the presence of neutralizing TNFR_2_-Ab (Fig. 3A). Preincubation with the cPLA_2_ inhibitor, methyl arachidonyl fluorophosphonate (MAFP), suppressed TNFα signaling whereas addition of the phospholipase A_2_ activating peptide (PLAP) mimicked TNFα effect, and stimulated Ca^2+^ influx (Fig. 3B). TNFα-induced activation was unaffected by the pretreatment with the cyclo-oxygenase inhibitor indomethacin but impaired in the presence of a lipoxygenase inhibitor nordihydroguaiaretic acid (NDGA), suggesting the potential requirement of AA metabolism into leukotrienes in this signal transduction (Fig. 3C). TNFα signaling persisted in the presence of the antagonist of leukotriene receptors, montelukast, suggesting an effect independent of binding to external receptors (Fig. 3C). These results indicate that TNFα signals in iso-hypertrophied cardiomyocytes through TNFR_2_ to activate cPLA_2_ and produce AA potentially leading to increased leukotriene levels which in turn activate Orai3.

**Figure 4:**
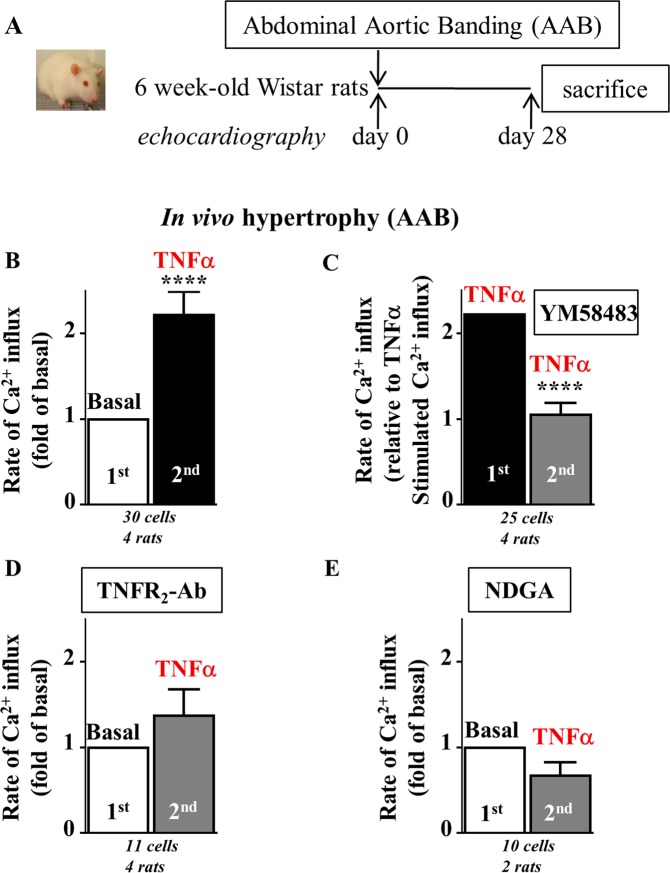
TNFα activates Orai-Ca^2+^ influx after binding to TNFR_2_ and potential production of AA metabolites via the lipoxygenase pathway in hypertrophied cardiomyocytes from rats with AAB-induced EACH. (**A**) Schematic representation of the protocol where rats with AAB-induced EACH for 28 days were subjected to echographic analyses. (**B**–**E**) Hypertrophied cardiomyocytes isolated from AAB rats were loaded with Fura_2_ before measurement of voltage- and store-independent Ca^2+^ influx. TNFα activates Ca^2+^ entry (**B**) in a manner sensitive to Orai inhibitor YM58483 (**C**), neutralizing TNFR_2_-Ab (**D**) or lipoxygenase inhibitor NDGA (**E**). Number of cells analyzed and number of cell isolations (rats) as indicated, mean ± SEM of cells, Wilcoxon matched-paired tests to examine if the mean of the 2^nd^ rate differs from the 1^st^ one, arbitrarily set as 1 (**B**,**D**,**E**) or 2.2 (**C**) ****p < 0.0001.

Of note, we recently demonstrated the emergence of an Orai3-dependent pathway that drives an AA-dependent Ca^2+^ influx in hypertrophied cardiomyocytes from rats subjected to abdominal aortic banding (AAB) for 28 days^12^. Complementary experiments were performed to examine the regulation of Orai3 by TNFα in this model (protocol in Fig. 4A). EACH remodeling was confirmed in AAB rats, relative to Sham rats, by an increase in IVSd, IVSs, PWd and PWs and concentric hypertrophy (h/r) (Table 2). AAB-induced hypertrophy was also attested by an increased cell area (4718 ± 128 *vs*. 3311 ± 124 µm^2^, n = 129 *vs*. 117 cells, *p* < 0.0001, Mann Whitney U test). A slight decrease of FS was observed in this model (Table 2).

**Table 2:** echocardiography parameters at day 28 in rats after AAB.

	d28 Sham rats (n = 7)	d28 AAB rats (n = 6)	Mann-Whitney U test
HR (bpm)	386 ± 12	383 ± 18	ns
IVSd (mm)	1.31 ± 0.03	2.08 ± 0.05	*p* < 0.001
LVd (mm)	7.43 ± 0.15	8.5 ± 0.4	*p* < 0.05
PWd (mm)	1.67 ± 0.03	2.38 ± 0.15	*p* < 0.01
IVSs (mm)	2.23 ± 0.1	2.7 ± 0.08	*p* < 0.05
LVs (mm)	4.07 ± 0.1	5.53 ± 0.3	*p* < 0.01
PWs (mm)	2.6 ± 0.05	3.2 ± 0.17	*p* < 0.05
h/r	0.41 ± 0.01	0.53 ± 0.04	*p* < 0.05
EF (%)	81.1 ± 0.6	70 ± 2.1	*p* < 0.001
FS (%)	44.7 ± 0.7	35.4 ± 1.5	*p* < 0.01

HR, heart rate; IVSd, end-diastolic interventricular septum thickness; LVd, end-diastolic left ventricular diameter; PWd, end-diastolic posterior wall thickness; IVSs, end-systolic interventricular septum thickness; LVs, end-systolic left ventricular diameter; PWs, end-systolic posterior wall thickness; h/r, diastolic wall thickness to radius ratio; EF, ejection fraction; FS, fractional shortening.

TNFα also induced activation of Orai3-dependent Ca^2+^ influx in AAB-induced hypertrophied cardiomyocytes (Fig. 4B,C) that relied on binding to TNFR_2_ (Fig. 4D) and potential production of AA metabolites via activation of the lipoxygenase pathway (Fig. 4E).

Thus, emergence of a TNFR_2_-dependent Orai3-driven Ca^2+^ influx characterized cardiomyocyte hypertrophy triggered by either iso-treatment or pressure overload.

### Inflammatory CD11b/c cells trigger TNFR_2_-dependent activation of Orai3-Ca^2+^ influx in hypertrophied cardiomyocytes

Next experiments aimed to evaluate the potential cellular source of inflammation in EACH hearts. Immunohistological examination of cardiac sections from iso-induced EACH rats indicated an increased number of TNFα-positive cells as compared to control and 66 ± 4% of TNFα-positive cells were identified as myeloid CD11b/c-positive cells (Fig. S2).

**Figure 5:**
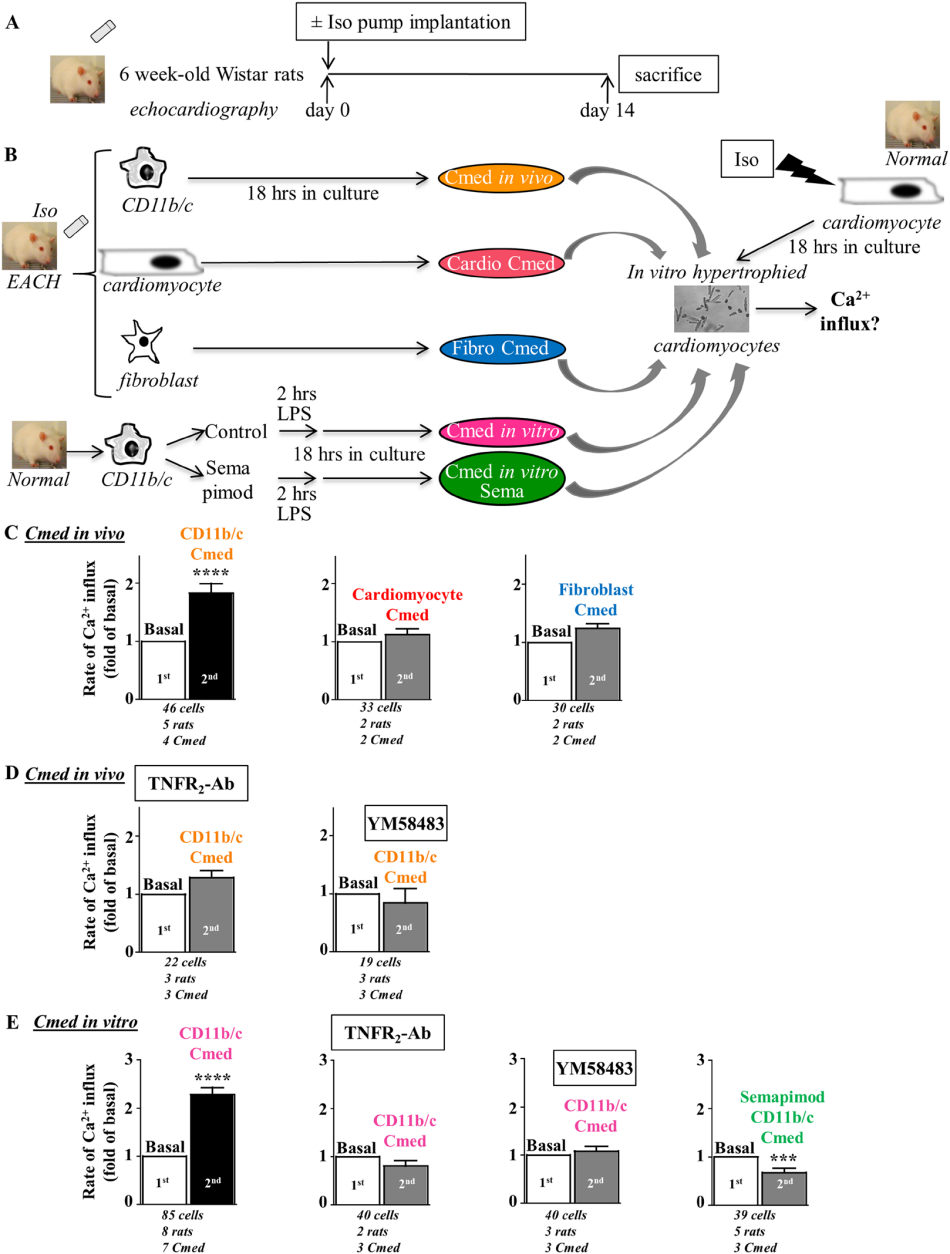
Conditioned medium from CD11b/c cells isolated from EACH heart activates Ca^2+^ influx in a manner sensitive to TNFR_2_-Ab and Orai inhibitor YM58483. (**A**) Schematic representation of the protocol where rats implanted with an iso-pump for fourteen days were subjected to echographic analyses and developed EACH. (**B**) CD11b/c cells, cardiomyocytes and fibroblasts were isolated from EACH hearts and cultured for 18 hours before recovery and concentration of conditioned media. CD11b/c cells pre-incubated with/without the anti-inflammatory drug, semapimod, prior *in vitro* LPS application to induce pro-inflammatory activation, were isolated from normal hearts and cultured for 18 hours before recovery and concentration of conditioned media. Iso-treated hypertrophied cardiomyocytes were loaded with Fura_2_ before measurement of voltage- and store-independent Ca^2+^ influx, 1^st^ in the absence and 2^nd^ in the presence of these conditioned media (Cmed). (**C**) Only Cmed from CD11b/c cells isolated from EACH hearts activates a voltage- and store-independent Ca^2+^ influx (**D**) in a manner sensitive to TNFR_2_-Ab and Orai inhibitor YM58483. (**E**) Cmed from CD11b/c cells isolated from normal hearts and *in vitro* stimulated with LPS activates a voltage- and store-independent Ca^2+^ influx in a manner sensitive to neutralizing TNFR_2_-Ab, Orai inhibitor YM58483 and anti-inflammatory pretreatment with semapimod. Number of cells analyzed, number of cell isolations (rats) and number of Cmed tested as indicated, mean ± SEM of cells, Wilcoxon matched-paired tests to examine if the mean of the 2^nd^ rate differs from the 1^st^ one, arbitrarily set as 1, ***p < 0.001, ****p < 0.0001.

Rat hearts (obtained from rats implanted with iso-pump for 14 days, Table 1) were used to isolate cardiomyocytes, fibroblasts and myeloid CD11b/c cells (denominated as *in vivo* cell activation) (Fig. 5A,B). Conditioned medium (Cmed) obtained from cardiomyocytes or cardiac fibroblasts were without effect on voltage- and store-independent Ca^2+^ influx in *in vitro* hypertrophied cardiomyocytes in contrast to the Cmed obtained from their cardiac CD11b/c counterparts (Fig. 5C). This pointed out the CD11b/c cells as the potential source of the Orai3 inflammatory trigger in EACH hearts. Of note, levels of TNFα detected in Cmed from CD11b/c cells (1.15 ± 0.3 pg/ml, n = 6) were 10 to 20 fold higher than levels measured in Cmed from their cardiac counterparts. CD11b/c-Cmed-induced activation was sensitive to neutralizing TNFR_2_ antibodies and Orai inhibitor YM58483 (Fig. 5D).

A similar effect was triggered using *in vitro* CD11b/c-Cmed (obtained from CD11b/c cells isolated from normal rat hearts and *in vitro* stimulated with lipopolysaccharide (LPS) (10 ng/ml for 2 hours) to induce pro-inflammatory activation) (Fig. 5B,E). When CD11b/c cells were pretreated with the anti-inflammatory drug semapimod (Sema) before LPS stimulation, *in vitro* CD11b/c-Cmed Sema was without effect on Ca^2+^ influx (Fig. 5B,E). Anti-inflammatory impact of semapimod was indicated by a reduction in TNFα-positive staining of CD11b/c cells (Fig. S3) and a limited TNFα content in Cmed Sema as compared to Cmed LPS (0.01 ± 0.004 *vs*. 0.24 ± 0.06 pg/ml, n = 5 *vs*. 8, *p* < 0.01, Mann Whitney U test).

These results highlighted the cardiac inflammatory CD11b/c cells as the potential triggers of Orai3-dependent Ca^2+^ influx in hypertrophied cardiomyocytes.

### TNFα or inflammatory cardiac CD11b/c cells trigger TNFR_2_ and Orai signaling pathways, enhancing hypertrophy and promoting resistance to oxidative stress in hypertrophied cardiomyocytes

**Figure 6:**
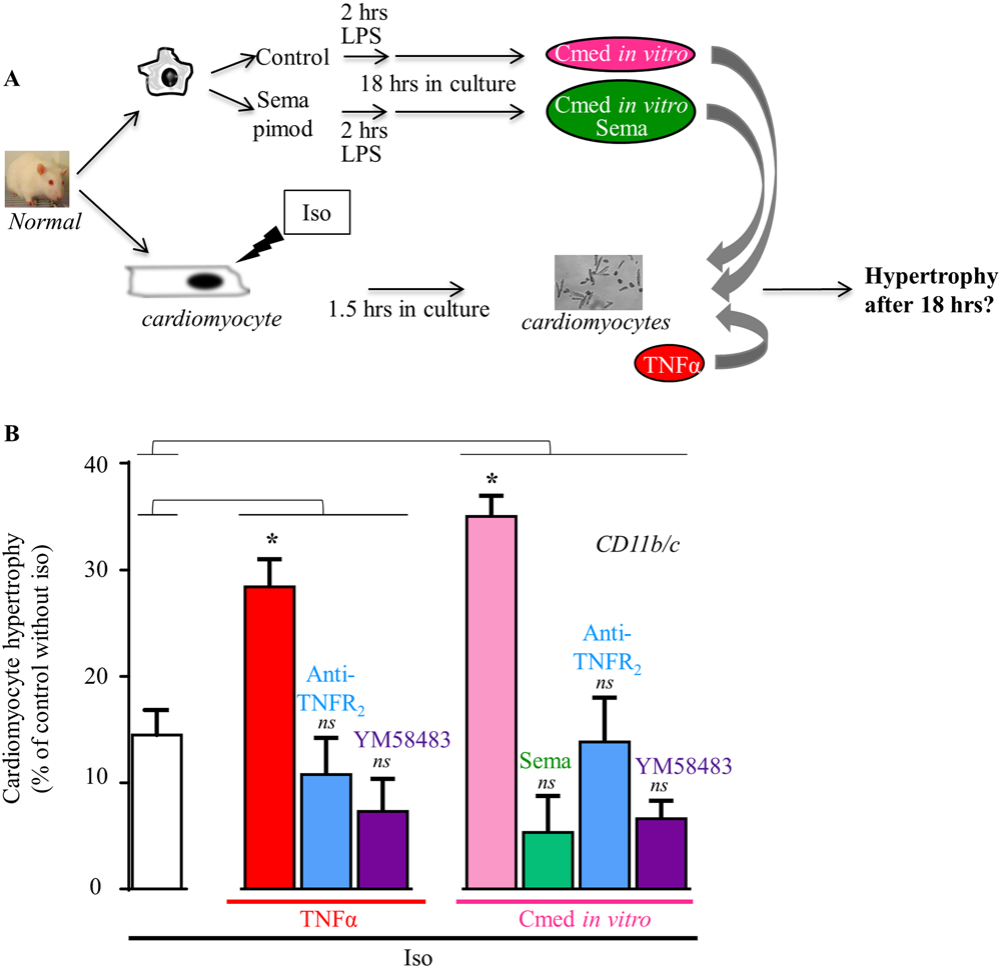
TNFR_2_ and Orai signaling pathways enhance hypertrophy in cardiomyocytes after inflammatory stimulation with TNFα or Cmed from *in vitro* LPS-activated cardiac CD11b/c cells. (**A**) CD11b/c cells were isolated from normal hearts and further subjected to *in vitro* activation by LPS incubation following or not semapimod pretreatment. Conditioned media (recovered after 18 hrs in culture) or TNFα were applied on rat cardiomyocytes after 1.5 hours iso treatment and cell hypertrophy analyzed after 18 hours. (**B**) TNFα or Cmed *in vitro* enhance hypertrophy of iso-treated cardiomyocytes in a manner sensitive to semapimod, TNFR_2_-Ab or YM58483. Mean ± SEM of 3–4 experiments, cardiomyocytes from 2–4 rats, 2 Cmed. Kruskal-Wallis followed by Dunn’s post-hoc test *vs*. the Iso-treated control (white column), *p < 0.05.

We next investigated the potential impact of inflammation on cardiomyocyte hypertrophy and evaluated the role of TNFR_2_ and Orai signaling pathways. Cardiomyocyte hypertrophy was initially induced by the iso stimulation (100 nM) for 1.5 hours. Cells were then challenged with TNFα or *in vitro* CD11b/c-Cmed. Cell hypertrophy was measured after 18 hours and attested by an increase in cell area (Fig. 6A). Addition of iso alone triggered a mean 14 ± 2% hypertrophy (Fig. 6B). TNFα enhanced iso-induced hypertrophy (up to 28 ± 3%), in a TNRF_2_- and YM58483-sensitive manner (hypertrophy reduced to 11 ± 3% and 7 ± 3%, by preincubation with TNFR_2_-Ab or YM58483, respectively) (Fig. 6B). CD11b/c-Cmed also increased iso-induced hypertrophy (up to 35 ± 2%) (Fig. 6B). Pretreatment of CD11b/c cells with anti-inflammatory semapimod blunted the prohypertrophic effect of Cmed (reduced to 5 ± 3%), as well as treatment with neutralizing TNFR_2_-Ab or YM58483 (reduced to 14 ± 4% and 7 ± 2%, respectively) (Fig. 6B).

These results indicate that inflammation (TNFα or Cmed from cardiac inflammatory CD11b/c cells) enhances cardiomyocyte hypertrophy via TNFR_2_- and Orai-dependent signaling pathways.

**Figure 7:**
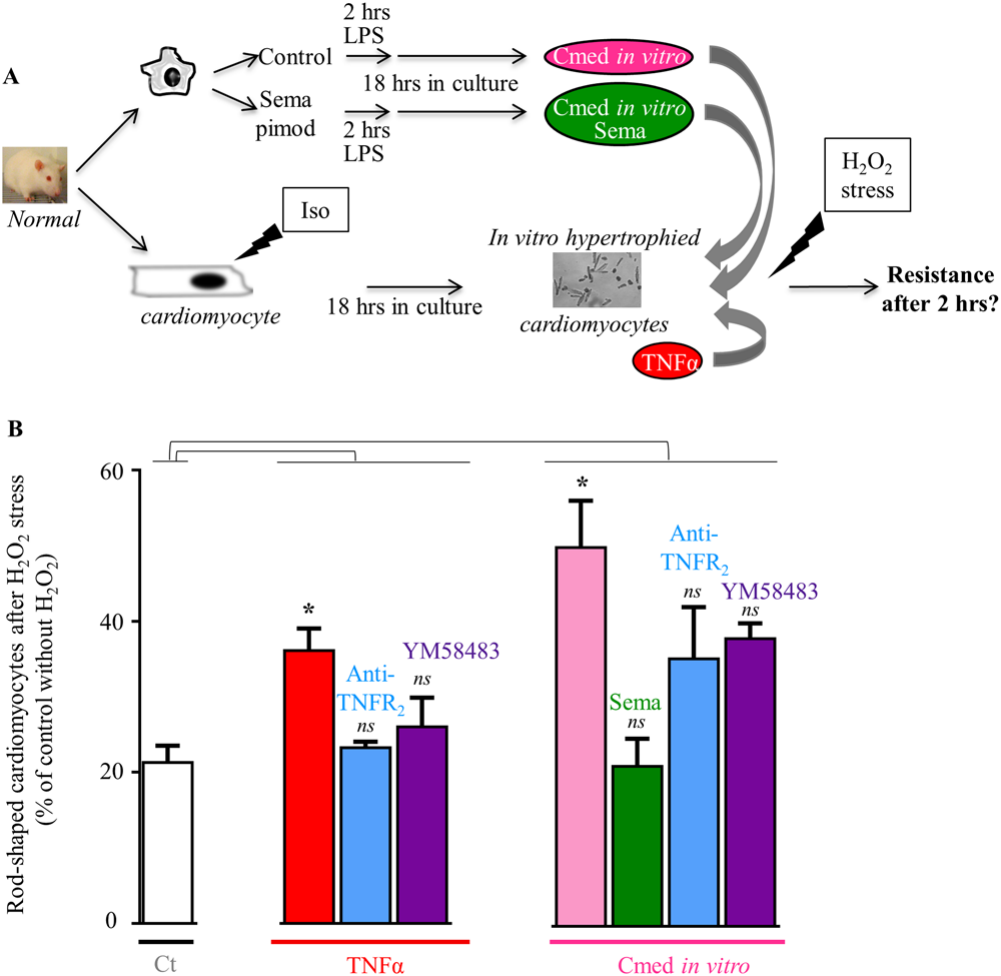
TNFR_2_ and Orai signaling pathways promote resistance to oxidative stress of cardiomyocytes after inflammatory stimulation with TNFα or Cmed from *in vitro* LPS-activated cardiac CD11b/c cells. (**A**) CD11b/c cells were isolated from normal hearts and further subjected to *in vitro* activation by LPS incubation following or not semapimod pretreatment. Conditioned media (recovered after 18 hrs in culture) or TNFα were applied on *in vitro* iso hypertrophied rat cardiomyocytes before H_2_O_2_ treatment and analyses of cell resistance. (**B**) TNFα or Cmed *in vitro* increase resistance of hypertrophied cardiomyocytes to H_2_O_2_ stress in a manner sensitive to semapimod, TNFR_2_-Ab or YM58483. Mean ± SEM of 3–5 experiments, cardiomyocytes from 3–5 rats, 2–4 Cmed. Kruskal-Wallis followed by Dunn’s post-hoc test *vs*. the Iso-treated control (white column), **p < 0.01.

We also evaluated the potential impact of inflammation on the resistance to oxidative stress using *in vitro* hypertrophied cardiomyocytes and demonstrated the role of TNFR_2_ and Orai signaling pathways. Iso-hypertrophied cardiomyocytes (100 nM for 18 hours) were preincubated with TNFα or *in vitro* CD11b/c-Cmed, and cell resistance was estimated by the number of rod-shaped cells after H_2_O_2_-oxidative stress (100 µM H_2_O_2_ for 2.5 hours) (Fig. 7A). Counting of resistant cells indicated 21 ± 2% *vs*. 100% in the presence and in the absence of H_2_O_2_, respectively (Fig. 7B). Treatment with TNFα increased resistance of hypertrophied cardiomyocytes from 21 ± 2% to 36 ± 3%, in a TNFR_2_- and YM58483-sensitive manner (rod-shaped cells reduced to 23 ± 1% and 26 ± 4%, after preincubation with the TNFR_2_-Ab or YM58483, respectively) (Fig. 7B). CD11b/c-Cmed alleviated the deleterious impact of H_2_O_2_, improving the yield of resistant cells to 50 ± 6% (Fig. 7B). Pretreatment of CD11b/c cells with anti-inflammatory semapimod blunted the beneficial impact of Cmed resulting in only 21 ± 4% resistant cells, as well as pretreatment with the TNFR_2_-Ab or YM58483 (36 ± 3% and 38 ± 2% resistant cells, respectively) (Fig. 7B).

These results demonstrate that inflammation (TNFα or Cmed from cardiac inflammatory CD11b/c cells) improves resistance to H_2_O_2_ in hypertrophied cardiomyocytes via TNFR_2_- and Orai-dependent protective signaling pathways.

Taken together our *in vitro* results argue for a protective Orai3-driven signal emerging during the phase of EACH and promoting adaptive cardiomyocyte hypertrophy and beneficial resistance to oxidative stress. In order to evaluate their relevance in the pathophysiology of HF, we performed a kinetic analysis of echocardiographic parameters and evaluated tissue remodeling in response to a unique intramyocardial Orai3 siRNA injection applied at the onset of EACH.

### Orai3 neutralization during EACH impairs cardiac hypertrophy, fosters alteration of function and dilation and deleterious tissue remodeling

To validate *in vivo* the protective role of Orai3 activity on EACH, we studied the effect of cardiac Orai3 knockdown during iso infusion in mice. First, as shown in Fig. S4, as compared to previous results obtained in rats (Table 1 and^12^), we checked that this model displayed similar iso-induced EACH remodeling after a 14 days infusion period in control mice. EACH was characterized by evolution of echocardiographic parameters, increase in heart weight to body weight ratio and cardiomyocyte size, and associated with an increase in Nppa and Tnfα mRNA expressions, but no change in Orai3 mRNA level (Fig. S4 and Table S2). This model was chosen to develop a novel approach of percutaneous intramyocardial injection of siRNA under echographic guidance validated in mice by other groups (i.e.^33^) in order to avoid potential artefactual consequences of the surgical thoracotomy on tissue remodeling that are susceptible to affect the post-operative echographic surveillance and evolution of cardiac parameters.

**Figure 8:**
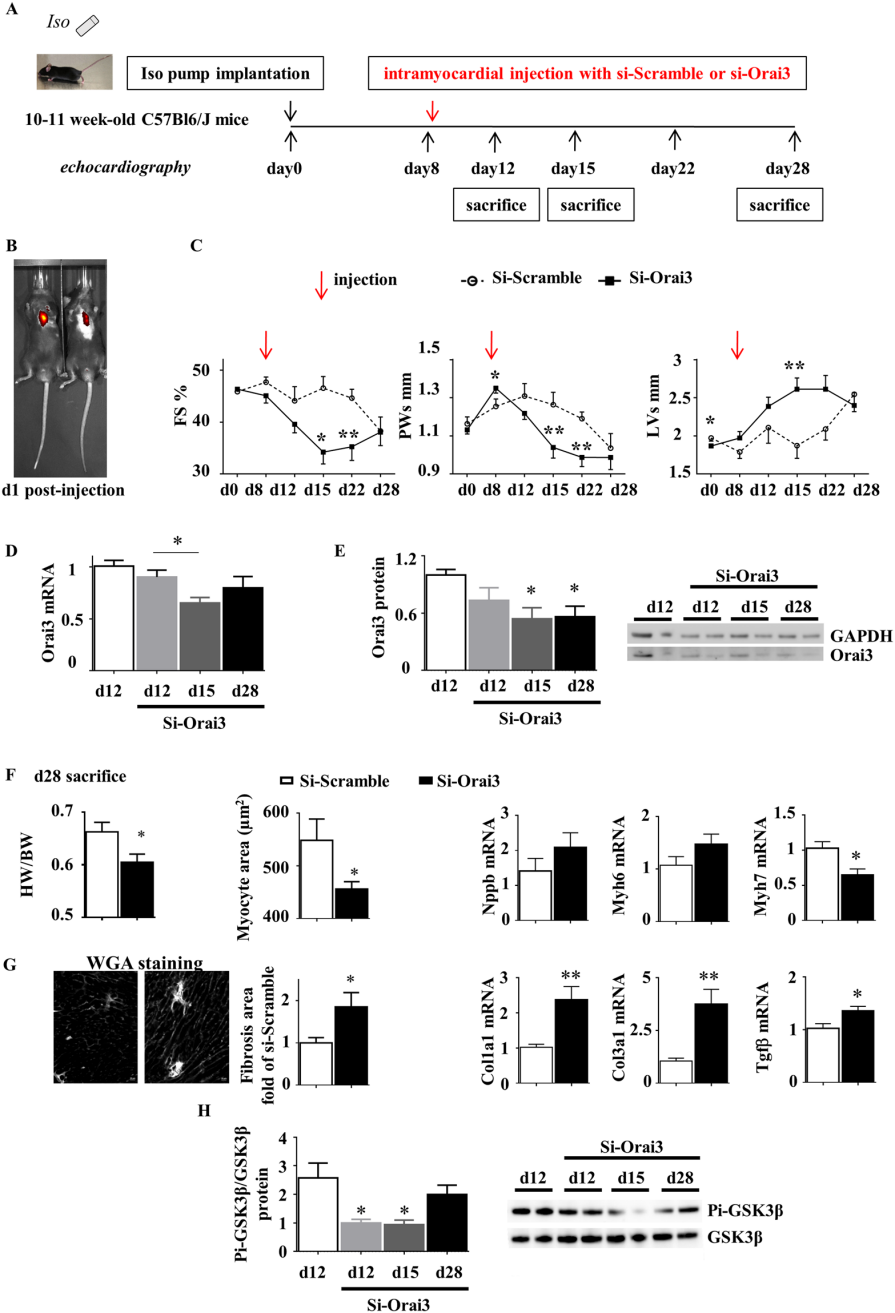
Orai3 knockdown at the onset of EACH limits cardiac hypertrophy, accelerates alteration of function and dilation and promotes fibrosis. (**A**) Schematic representation of the protocol where mice implanted with an iso-pump at day 0 were subjected to a unique ultrasound-guided intramyocardial transthoracic injection of Scramble or Orai3 siRNA at day 8 and submitted to an echocardiographic follow-up. (**B**) In some experiments, siRNA was added with sulforhodamine: typical *in-vivo* visualization of intracardiac localization of sulforhodamine at day 1 post-injection using an IVIS spectrum *in-vivo* imaging system. (**C**) Echocardiographic parameters (mean ± SEM of mice, n = 8–22 mice/group, see Table 3, ANOVA for repeated measures followed by Dunn-Sidak post-hoc tests). (**D**,**E**) Efficient knockdown of Orai3 mRNA and protein levels in cardiac homogenates at d12, d15 and d28 following injection (mean ± SEM of mice, n = 7 mice/group, Kruskal-Wallis followed by Dunn’s post-hoc tests). Full length blots were included in SI. (**F**,**G**) SiOrai3 injection induced a decrease in Heart Weight/Body Weight ratio, myocyte area and Myh7 mRNA level, an increase in fibrosis, and an elevation of Col1a1, Col3a1 and Tgfβ mRNA levels (mean ± SEM of mice, n = 7 mice/group, Mann-Whitney U tests). (**H**) SiOrai3 injection induced a decrease in the ratio phospho-GSK3β/GSK3β at day 12 and day 15 following injection (mean ± SEM of mice, n = 7 mice/group, Kruskal-Wallis followed by Dunn’s post-hoc test). Full length blots were included in SI. *p < 0.05, **p < 0.01.

Iso-infused mice were subjected to a unique intramyocardial injection of either Scramble or Orai3 siRNA at day 8 following pump implantation (Fig. 8A). Efficient knockdown of Orai3 during EACH was attested at the mRNA and protein levels (Fig. 8D,E). At day 15, hearts from siOrai3-injected mice displayed a decreased hypertrophy (lower PWs), an increased dilation (higher LVs) and an altered cardiac function (lower FS) as compared to hearts from siScramble-injected mice (Fig. 8C and Table 3). At day 28, siOrai3-injected mice still presented with a lower heart weight to body weight ratio, a smaller cardiomyocyte area, a decrease in Myh7 mRNA level, as compared to siScramble-injected mice (Fig. 8F). Orai3 knockdown during EACH also fostered fibrosis attested by histological analysis and increased Col1a1, Col3a1 and Tgfβ mRNA levels (Fig. 8G). Mechanistically, a decrease in the ratio phospho-GSK3β/GSK3β was correlated with the reduction of Orai3 expression (Fig. 8H).

**Table 3:** kinetics of echocardiography parameters in mice implanted with Iso pump after Scramble (Scr) or Orai3 siRNA intramyocardial injection.

Iso mice (iso pump implantation at d0)																		
Time of echocardio-graphy	d0			d8			d12			d15			d22			d28		
Injection at d8	Scr (n = 11)	Orai3 (n = 22)	*p*	Scr (n = 11)	Orai3 (n = 22)	*p*	Scr (n = 11)	Orai3 (n = 22)	*p*	Scr (n = 11)	Orai3 (n = 15)	*p*	Scr (n = 8)	Orai3 (n = 8)	*p*	Scr (n = 8)	Orai3 (n = 8)	*p*
HR (bpm)	624 ± 7	634 ± 3	*ns*	668 ± 11	652 ± 9	*ns*	606 ± 15	619 ± 9	*ns*	622 ± 11	632 ± 12	*ns*	630 ± 6	609 ± 8	*ns*	594 ± 29	618 ± 11	*ns*
IVSd (mm)	0.6 ± 0.01	0.7 ± 0.01	*ns*	1 ± 0.02	1 ± 0.02	*ns*	1 ± 0.06	0.9 ± 0.03	*ns*	1 ± 0.04	0.8 ± 0.03	<*0.05*	0.9 ± 0.04	0.8 ± 0.03	*ns*	0.8 ± 0.04	0.7 ± 0.04	*ns*
LVd (mm)	3.6 ± 0.05	3.5 ± 0.04	<*0.05*	3.4 ± 0.11	3.6 ± 0.07	*ns*	3.7 ± 0.19	3.9 ± 0.09	*ns*	3.4 ± 0.17	4.0 ± 0.10	<*0.05*	3.8 ± 0.18	4.0 ± 0.14	*ns*	4.1 ± 0.24	3.8 ± 0.14	*ns*
PWd (mm)	0.7 ± 0.02	0.7 ± 0.01	*ns*	0.9 ± 0.03	0.9 ± 0.02	*ns*	0.9 ± 0.05	0.8 ± 0.02	<*0.05*	0.9 ± 0.05	0.7 ± 0.04	<*0.01*	0.8 ± 0.02	0.7 ± 0.03	*ns*	0.7 ± 0.05	0.6 ± 0.03	*ns*
IVSs (mm)	1.1 ± 0.02	1.1 ± 0.02	*ns*	1.5 ± 0.03	1.5 ± 0.04	*ns*	1.4 ± 0.07	1.4 ± 0.04	*ns*	1.5 ± 0.06	1.1 ± 0.05	<*0.05*	1.4 ± 0.06	1.3 ± 0.04	*ns*	1.3 ± 0.06	1.3 ± 0.07	*ns*
LVs (mm)	2.0 ± 0.04	1.9 ± 0.02	<*0.05*	1.8 ± 0.08	2.0 ± 0.08	*ns*	2.1 ± 0.19	2.4 ± 0.12	*ns*	1.9 ± 0.16	2.6 ± 0.14	<*0.01*	2.2 ± 0.17	2.6 ± 0.17	*ns*	2.6 ± 0.29	2.4 ± 0.16	*ns*
PWs (mm)	1.2 ± 0.03	1.1 ± 0.02	*ns*	1.3 ± 0.04	1.4 ± 0.02	<*0.05*	1.3 ± 0.06	1.2 ± 0.03	*ns*	1.3 ± 0.06	1.0 ± 0.05	<*0.01*	1.2 ± 0.03	1.0 ± 0.04	<*0.01*	1.0 ± 0.07	1.0 ± 0.06	*ns*
h/r	0.3 ±0.007	0.3 ±0.004	*ns*	0.56 ±0.018	0.5 ±0.017	*ns*	0.54 ±0.044	0.4 ±0.016	<*0.05*	0.55 ±0.041	0.40 ±0.021	<*0.01*	0.44 ±0.024	0.39 ±0.019	*ns*	0.38 ±0.042	0.36 ±0.019	*ns*
EF (%)	83.1 ± 0.5	83.5 ± 0.2	*ns*	84.6 ± 0.8	81.7 ± 1.6	*ns*	80.0 ± 2.6	75.7 ± 2.1	*ns*	82.8 ± 2.2	68.5 ± 3.2	<*0.05*	79.4 ± 1.9	70.6 ± 3	<*0.01*	72.7 ± 4.8	74.1 ± 2.7	*ns*
FS (%)	45.9 ± 0.5	46.3 ± 0.2	*ns*	47.7 ± 0.9	45.1 ± 1.4	*ns*	44.1 ± 2.6	39.6 ± 1.6	*ns*	46.5 ± 2.1	34.2 ± 2.2	<*0.05*	44.6 ± 1.6	35.3 ± 2.4	<*0.01*	38.3 ± 2.6	38.1 ± 2.4	*ns*

Two-way ANOVA followed by Sidak’s post-hoc tests.

HR, heart rate; IVSd, end-diastolic interventricular septum thickness; LVd, end-diastolic left ventricular diameter; PWd, end-diastolic posterior wall thickness; IVSs, end-systolic interventricular septum thickness; LVs, end-systolic left ventricular diameter; PWs, end-systolic posterior wall thickness; h/r, diastolic wall thickness to radius ratio; EF, ejection fraction; FS, fractional shortening.

Importantly, the intramyocardial injection of Orai3 siRNA in control mice showed that knockdown of Orai3 was without functional impact (Fig. S5 and Table S3).

Taken together, these results demonstrated the emergence of a functional protective role of Orai3 during EACH.

## Discussion

**Figure 9:**
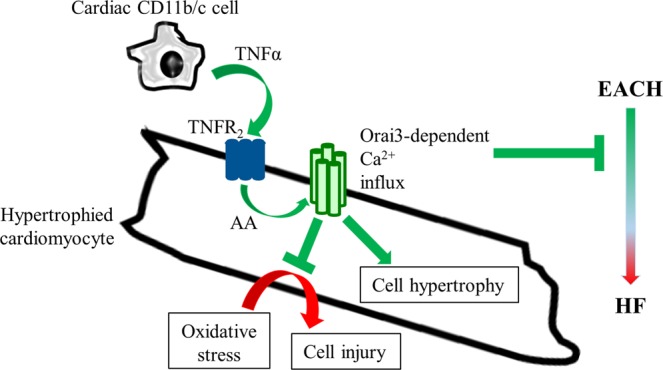
Orai3 enhances hypertrophy, promotes resistance to oxidative stress in adult hypertrophied cardiomyocytes and during EACH limits evolution towards HF. Mechanistically, a TNFα paracrine signaling potentially mediated by the cardiac inflammatory CD11b/c cells promotes TNFR_2_-dependent activation of store-independent arachidonic acid-dependent Orai3-related Ca^2+^ influx enhancing hypertrophy and inducing resistance to H_2_O_2_. Knockdown of Orai3 at the onset of adaptive hypertrophy suppresses the EACH response and accelerates transition towards HF.

Our results highlight the emergence of a protective role for Orai3 in the hypertrophied cardiomyocytes. We identified TNFα as a mechanistic trigger of the TNFR_2_-dependent activation of Orai3-Ca^2+^ influx and showed that CD11b/c cells are a potential driving source of this signaling in EACH hearts. This paracrine signaling enhances hypertrophy and promotes resistance to oxidative stress of hypertrophied cardiomyocytes. Furthermore, we show that Orai3 knockdown during EACH fosters HF (Fig. 9).

Cardiac Orai3-dependent Ca^2+^ influx was previously identified as a prohypertrophic stimulus in AAB-induced CH^12^. We confirm these results in a model of iso-induced EACH, a model of reproducible progressive concentric hypertrophy. In our study, we found that *in vivo* reduction in Orai3 expression in iso-infused rats decreased the mean cardiomyocyte area. In keeping with this atrophic impact of Orai3 siRNA, neutralization of Orai3 during EACH in iso-infused mice triggers a rapid and significant decrease in hypertrophic parameters still detectable at day 20 post injection. Furthermore, our *in vitro* experiments document a direct prohypertrophic effect of Orai3 in isolated iso-treated cardiomyocytes.

Interestingly, our results highlight novel protective properties of Orai3-dependent Ca^2+^ influx. In line with the reported resistance of Orai3 channel to redox regulation^34^, we show that Orai3 activation confers resistance to oxidative stress in isolated hypertrophied cardiomyocytes. Furthermore, our *in vivo* results indicate that efficient cardiac knockdown of Orai3 during EACH inhibits adaptive hypertrophy, alters cardiac function and promotes fibrosis. Mechanistically, recent results from our laboratory indicate that the Orai3-interacting protein STIM1 is essential to tune the Akt/GSK3β prosurvival signaling^8–10,35,36^. Our *in vivo* results show that neutralization of Orai3 during EACH is associated with a decrease in phospho-GSK3β/GSK3 ratio. In the current absence of published knockout data for Orai3, our study is the first to address its pathophysiological relevance and indicate its essential protective role during EACH.

We have identified the first pathophysiological trigger of Orai3-driven store-independent Ca^2+^ influx in cardiomyocytes, namely a TNFα/TNFR_2_-dependent signaling. Interestingly, the regulation of Orai3 by TNFα is detected in hypertrophied cardiomyocytes (both in response to iso treatment or in the AAB-model), but not in normal cardiomyocytes. Of note, iso-hypertrophied and normal cardiomyocytes display similar TNFR_2_ expression (2.4 ± 0.4 *vs*. 2.4 ± 0.3 pg TNFR_2_/mg, respectively, n = 4). However, and as previously reported in AAB-induced CH hearts^12^, co-immunoprecipitation experiments using STIM1 antibodies indicate enhanced Orai3 recruitment to STIM1 in the iso-induced EACH (Fig. S6). Since Orai3-STIM1 interaction is a prerequisite for Orai3-dependent Ca^2+^ influx activation^14,17^, its absence in normal cells and its enhancement in hypertrophied cells constitute a major difference likely to explain the lack of impact of Orai3-dependent Ca^2+^-influx as well as the absence of TNFα regulation in normal cells. Accordingly, our *in vivo* and *in vitro* experiments in normal mice show that the knockdown of Orai3 is without impact on echocardiographic parameters as well as heart or cardiomyocyte size. They argue for a pathophysiological impact of Orai3 exerted during EACH but not under control conditions. Dominant impact of inflammation is currently reported as deleterious in normal hearts, as it is associated with reactive oxygen species production, proapoptotic signaling and a dominant role of TNFR_1_ pathways overwhelming TNFR_2_ signaling^26^. Using TNFR_1_ and TNFR_2_ knockout mice implanted with iso-pumps, Prabhu *et al*. demonstrated that TNFR_2_ but not TNFR_1_ signaling prevents the detrimental long-term effects of β-adrenergic receptor stimulation in the heart^37^. In another study, the group of Prasad proposed that the beneficial effects of TNFR_2_ signaling in presence of sympathetic overdrive could act through the preferential TNFR_2_-mediated recruitment of GRK2 to mediate βAR desensitization thus reducing deleterious cardiac signaling and remodeling. In agreement with these studies, our study further suggests the emergence of a protective TNFR_2_ pathway during EACH development via the stimulation of a novel downstream effector Orai3.

We show here that cardiac CD11b/c cells are a potential source of inflammatory TNFα/TNFR_2_-dependent signaling leading to Orai3-dependent Ca^2+^ channel activation. Both Cmed from *in vitro* and *in vivo* activated CD11b/c cells stimulate a store-independent Ca^2+^ influx in hypertrophied cardiomyocytes in a TNFR_2_-Ab and YM58483 sensitive manner. This suggests the presence of activated inflammatory CD11b/c cells in EACH hearts. We demonstrated that LPS-activated CD11b/c cells enhance hypertrophy and promote resistance of hypertrophied cardiomyocytes to oxidative stress, in a TNFR_2_-Ab and YM58483 sensitive manner. This protective impact is blunted by preincubation of CD11b/c cells with the anti-inflammatory drug semapimod. Our data strengthen the concept that inflammation arising from cardiac myeloid cells may exert paracrine beneficial impact on cardiomyocytes^38,39^. Of note, cardiac macrophages are an emerging focus for therapeutic strategies aimed at minimizing cardiomyocyte death, ameliorating pathological cardiac remodeling and for treating HF^40^.

We observed that TNFα-dependent activation of Orai3-Ca^2+^ influx relies on cPLA_2_ activation and is mimicked by a cPLA_2_ activator. This is in accordance with the reported presence of the lipid interaction site located in the NH_2_ terminal intracytosolic sequence of Orai3^20^. TNFα-dependent effect is sensitive to NDGA treatment which suggests the potential requirement of a lipoxygenase-dependent AA metabolism to activate Orai3-Ca^2+^ influx. Accordingly, Trebak *et al*. previously described LTC4, a lipoxygenase AA-metabolite, as an activator of Orai3 in the vascular smooth muscle cells^41^. However, this proposal needs to be tempered since NDGA, in addition to inhibit lipoxygenase activity may also exert several off target effects (i.e., PKC inhibition, overall anti-oxidant, ER-Golgi protein shuttling inhibition). Our results further illustrate the dual role of cPLA_2_-AA signaling in mediating TNFα effects in adult cardiac myocytes. We have identified Orai3 as a novel protective TNFα-cPLA_2_-AA pathway. Accordingly, the beneficial impact of TNFα-cPLA_2_-AA pathways has been previously reported on the cardiomyocyte calcium transients and contraction (i.e. by the group of Oceandy^42^ and our previous results^26,30^) and on the survival to oxidative stress^26^. In contrast, the group of Gugiyama reported the deleterious impact of a TNFα-cPLA_2_ cardiac signaling in a model of ischemia-reperfusion^43^.

In conclusion, our *in vitro* and *in vivo* studies characterize the Orai3 signaling pathway that exerts a direct protective role against HF in hypertrophied cardiomyocytes. Mechanistically, we have identified Orai3 as a novel driver of TNFR_2_-dependent inflammation instrumental in this protection. Furthermore, we showed a protective Orai3-dependent paracrine role of cardiac myeloid cells leading to adaptive hypertrophy and improved resistance to oxidative stress. In summary, our results are the first to address the functional role of Orai3 signaling in HF that may open new perspectives for patients’ treatments.

## Methods

### Ethics

Care of the animals and surgical procedures were performed according to the Directive 2010/63/EU of the European Parliament, which had been approved by the Ministry of Agriculture, France, (authorization for surgery C-75-665-R). The project was submitted to the French Ethic Committee CEEA (*Comité d’Ethique en Expérimentation Animale*) and obtained the authorization Ce5/2012/050 and APAFIS#1729-2015-083114195840v8. All experiments were performed in accordance with relevant named guidelines and regulations.

### Animals

6 week-old male Wistar rats and 10-11 week-old male C57BL/6JRj mice were purchased from Janvier Labs.

### *In vivo* chronic isoproterenol infusion

Rats and mice anesthetized under isoflurane (Iso-vet®, Piramal, UK) (1–3%) were implanted subcutaneously with an osmotic minipump (Alzet, Charles River) containing either isoproterenol (1.5 mg/kg/day for rat or 30 mg/kg/day for mouse) (iso-pump) or vehicle for 14 or 28 days to develop either EACH or HF, respectively, or as otherwise stated.

### Abdominal aortic banding

Rats were anaesthetized by intra-peritoneal injection of ketamine (Imalgene®, Merial, Germany) and xylazine (Rompun®, Bayer, Germany) (75 and 10 mg/kg, respectively). Medial abdominal laparotomy was performed and a clip with an internal opening of 0.58 mm was placed, as previously reported^12^. Sham-operated rats served as controls.

### Measurement of cardiac parameters

Echocardiography was performed on lightly anesthetized animals under isoflurane (0.5–1%) with a probe emitting ultrasounds from 8- to 14-MHz frequency (Vivid7 PRO apparatus; GE Medical System Co). The two-dimensionally guided Time Motion mode recording (parasternal long-axis view) of the left ventricle (LV) provided the following measurements: end-diastolic and end-systolic interventricular septum (IVSd and IVSs), posterior wall thicknesses (PWd and PWs), internal diameter (LVEDD and LVESD), and heart rate (HR). Each set of measurements was obtained from the same cardiac cycle. At least three sets of measurements were registered from three different cardiac cycles. Fractional shortening (FS): [(LVEDD − LVESD)/LVEDD] × 100 and h/r: [left ventricle diastolic wall thickness/radius] were calculated.

### *In vivo* ultrasound mediated Cy3-tagged siRNA delivery in rats

After an intra-peritoneal injection of ketamine and xylazine (75 and 10 mg/kg, respectively), an incision was made in the fourth inter-costal space to expose the heart and Cy3-tagged siRNAs were delivered as previously described^44^ two weeks after iso-pump implantation and three days before cardiac cell isolation. The siRNA for scramble (Life Technologies) and Orai3, were chosen from^12,45,46^ (FWsiOrai3: 5′-GUUUAUGGCCUUUGCCCUATT-3′; RVsiOrai3: 5′-UAGGGCAAAGGCCAUAAACTT-3′).

### *In vivo* intramyocardial ultrasound-guided transthoracic siRNA delivery in mice

On-target plus Scramble and Orai3 siRNA (Dharmacon GE healthcare) were injected by ultrasound-guided transthoracic intramyocardial injection, as described in^33^, at day 8 after iso-pump implantation. Echocardiographic parameters were measured regularly as stated.

### Cardiac cell isolation and culture

Rat cardiac myocytes were isolated either from chronically iso-infused hearts or from normal hearts, after injection or not with Cy3-tagged siRNA, three days before, when stated. Rats were administered a sodium pentobarbital (Ceva Sante Animale, France) intra-peritoneal injection (200 mg/kg). Hearts were harvested and kept in ice-cold Krebs-Henseleit (KH) solution supplemented with 10 mmol/L taurine and 0.5 mmol/L EGTA, then rapidly canulated and mounted on the Langendorff apparatus. The hearts were retrogradely perfused through the aorta, first with a Krebs-Henseleit (KH) solution supplemented with 10 mmol/L taurine for 5 minutes, then with enzymatic solution for 20 minutes. The KH solution contained (in mmol/L): 100 NaCl, 4 KCl, 5.5 NaHCO_3_, 1 KH_2_PO_4_, 1.7 MgCl_2_, 10 D-glucose, 15 2,3-butanedione monoxime, 22 Hepes, pH = 7.4 with NaOH. The enzyme solution was supplemented with 1 mg/ml collagenase A (Roche Applied Science, Meylan, France) and 5 mg/ml bovine serum albumin BSA (Sigma, Lyon, France). All chemicals were from Sigma (Lyon, France. Cell suspension was then used to differentially isolate ventricular cardiomyocytes, cardiac fibroblasts and cardiac CD11b/c cells.

Ventricular cardiomyocytes from iso-pump rats (*in vivo* hypertrophy) were plated onto laminin-coated glasses and maintained overnight in M199 medium (Life technologies). Cells isolated from normal hearts or from saline-pump rats were added with 100 nM isoproterenol plus 100 µM ascorbic acid (Sigma), when stated, to induce *in vitro* hypertrophy, three hours after plating, and let overnight in M199 medium.

Cardiac fibroblasts were isolated by centrifugation, plated onto 12-well plates and maintained in DMEM medium (Life technologies).

Cardiac CD11b/c cells were isolated by centrifugation, enriched using an anti-CD11b/c antibody coupled to magnetic beads (MiltenyiBiotec) and maintained overnight in RPMI medium (Life technologies) supplemented with 10 mmol/L Hepes. When stated, CD11b/c cells were *in vitro* polarized towards a pro-inflammatory phenotype upon incubation for 2 hours with 10 ng/ml lipopolysaccharide (LPS) before overnight incubation with a new LPS-free medium.

All cardiac cells were cultured for 18 hours following plating. Conditioned media from cardiac cells were concentrated on Amicon 10 kDa centrifugal filter.

### Fura-2 AM calcium imaging

Isolated rat ventricular myocytes were loaded with Fura_2_-AM (Molecular Probes, Life Technologies) as reported^26^. Transfected cells (detected by fluorescence imaging as cells positive for Cy3-tagged siRNA) or non-transfected cells, rhythmically beating in response to electrical stimulation (square waves, 0.5 Hz, as previously described^26^), were analyzed. Measurements were recorded on a Zeiss Platform equipped with an Axio Observer Z1 microscope, a DGA plus illuminator and a camera Coolsnap HQ2 (workstation Carl Zeiss).

Cells were first paced for few cycles and Ca^2+^ transients were recorded to ensure viability and functionality of the cell. Cells were incubated in tyrode buffer (1.8 mmol/L Ca^2+^) to check the stability of basal cytosolic calcium level and then switched to appropriate store- and voltage-independent Ca^2+^-free buffer. Store-independent Ca^2+^ entry was then measured upon readdition of 1 mmol/L Ca^2+^. Ca^2+^off/Ca^2+^on protocols repeated twice allowed paired comparison between two similar (for reproducibility assessment) or distinct perfusion conditions.

Tyrode buffer contained (in mmol/L): 135 NaCl, 4 KCl, 1 MgCl_2_, 10 D-glucose, 20 Hepes, pH = 7.4 with NaOH. Store- and voltage-independent buffer contained 1 µM ryanodine, 20 µM diltiazem and 135 mmol/L N-methyl D-glucamine (NMDG) instead of NaCl. All chemicals, excepted ryanodine (Tocris), were from Sigma.

Data analysis was performed using the Zen Software (2012, blue edition). The rates of Ca^2+^ entry were estimated by the slope of the first minute of initial increase in Fura-2 fluorescence ratios in response to the re-addition of Ca^2+^. 10–100 myocytes isolated from 2–16 animals were analyzed per experimental condition (as stated in Figs).

### Measurement of cell hypertrophy

Cardiomyocyte hypertrophy was estimated after 18 hours in culture in the presence of isoproterenol. After an initial incubation with iso alone (100 nM) for 1.5 hours, cardiomyocytes were then treated or not for 1 hour with TNFR_2_-Ab or YM58483 before addition of control medium, TNFα or CD11b/c Cmed. Cardiomyocytes were visualized using brightfield at x20 magnification and cell area was measured in at least 300 cells per condition per experiment. Results were the mean of at least three different experiments performed on two cell isolations (using at least two different *in vitro* CD11b/c-Cmed).

### Measurement of cell resistance to H_2_O_2_

Cell resistance experiments were performed as previously described^26^. *In vitro* hypertrophied cardiomyocytes were preincubated for 1 hour with or without TNFR_2_-Ab or for 10 minutes with or without YM58483. Then, TNFα or Cmed from *in vitro* activated CD11b/c cells, or control medium were added for 10 minutes before subsequent treatment or not with H_2_O_2_ (100 µM, (Sigma)) for 2.5 hours, that was the time corresponding to a mean 75% injury in response to H_2_O_2_ for control. Cardiomyocytes were visualized using brightfield at x100 magnification, and resistance was estimated by counting rod-shaped cells in 12 random microscopic fields. At least 300 cells were counted in each dish, and results were the mean of at least three different experiments performed on three different cell isolations (using at least two different *in vitro* CD11b/c-Cmed).

### Quantitative RT-PCR

Rat total RNA was isolated with the RNeasy Mini kit (Qiagen). RNA reverse transcriptase-PCR analysis was performed using the Absolute QPCR SYBR green mix (ABgene) on an MX3005P QPCR system (Stratagene, Agilent Technologies). Primer sequences are listed below in Table 4. Transcript levels were normalized to the RPL32 (rat) mRNA levels.

**Table 4:** Sequences of the primers used (5′ to 3′) for rat.

Orai3 (rat)	Fw 5′-CTGTCCACCAGTCACCACAC-3′
	Rv 5′-CCACCAAGGATCGGTAGAAA-3′
Ribosomal protein L32 (RPL32) (rat)	Fw 5′-CCAGAGGCATCGACAACA-3′
	Rv 5′-GCACTTCCAGCTCCTTGACAT-3′

Mice total RNA was isolated using TRIzol (invitrogen). RNA reverse transcriptase-PCR analysis was performed using Brilliant III Ultra-Fast SYBR® Green QPCR Master Mix (Agilent Technologies) on an LightCycler® 480 Real-Time PCR System (Roche). Primer sequences are listed below in Table 5. Transcript levels were normalized to the RPL13 (mice) mRNA levels.

**Table 5:** Sequences of the primers used (5′ to 3′) for mouse.

Nppb (mouse)	Fw 5′-CTGAAGGTGCTGTCCCAGAT-3′
	Rv 5′-CAGCAGCTTCTGCATCTTGA-3′
Col1a1 (mouse)	Fw 5′-CTCAGGGTGCTCGTGGAT-3′
	Rv 5′-CTTAGGACCAGCAGGACCAG-3′
Col3a1 (mouse)	Fw 5′-GATCTCCTGGTTCTCCTGGAT-3′
	Rv 5′-TCGTCCAGGTCTTCCTGACT-3′
Orai3 (mouse)	Fw 5′-GCCACCTCCTGTAAGCTCTG-3′
	Rv 5′-TCCTGGAGGAGCAAACAACT-3′
Myh6 (mouse)	Fw 5′-CCAAGTTCGACAAGATCGAG-3′
	Rv 5′-CCGAGTAGGTATAGATCATC-3′
Myh7 (mouse)	Fw 5′-AGCAGGTGGATGATCTGGAG-3′
	Rv 5′-CCAACTGCTGCTTGTCATTC-3′
Tgfβ (mouse)	Fw 5′-CTGAACCAAGGAGACGGAAT-3′
	Rv 5′-GGCTGATCCCGTTGATTTC-3′
Ribosomal protein L13 (Rpl13) (mouse)	Fw 5′-GAGGAGGCGAAACAAGTCCA-3′
	Rv 5′-GGGTGGCCAGCTTAAGTTCT-3′

### Western-blot

Cardiac myocytes were lysed in 150 mM NaCl, 50 mM Tris pH = 7.5, EDTA 5 mM, 0.5% NP40, 1% triton, protease and phosphatase inhibitors cocktail (Sigma, Lyon, France) and samples isolated by centrifugation. Forty µg of proteins in a final volume of thirty µL of Laemmli loading buffer were heated to 70 °C for 10 minutes. Samples were run on a 4–12% Nu-PAGE gel (Life technologies), transferred to Hybond-C PVDF membrane according to the manufacturer protocol (Amersham Biosciences, GE Healthcare). Membrane was incubated with rabbit anti-Orai3 (1/500, ProsciInc 4117), or rabbit anti-GAPDH (1/2500, Cell Signaling 2118) followed by anti-rabbit HRP (1/5000, Amersham Biosciences, GE Healthcare). Detection was performed using the ECL Western Blotting Substrate (Pierce) and signals were recorded using a Camera LAS 4000.

Cardiac tissue was lysed in 150 mM NaCl, 50 mM Tris pH = 7.5, EDTA 5 mM, 0.5% NP40, 1% triton, protease and phosphatase inhibitors cocktail (Sigma, Lyon, France) and samples isolated by centrifugation. Eighty µg of proteins in a final volume of fifteen µL of Laemmli loading buffer were heated to 70 °C for 10 minutes. Samples were run on a 4–12% Nu-PAGE gel (Life technologies), transferred to Trans-Blot Turbo Mini-size nitrocellulose membrane according to the manufacturer protocol (Bio-Rad). Membrane was incubated with rabbit anti-Orai3 (1/500, ProsciInc 4117), rabbit anti-Pi-GSK3β (1/1000, Cell signaling mAb#9323), rabbit anti-GSK3β (1/1000, Cell signaling mAb#9315), or mouse-GAPDH (1/1000, Santa Cruz sc-365062) followed by anti-rabbit HRP (1/10000, Abcam 6721), or anti-mouse HRP (1/5000, NA931 GE Healthcare). Detection was performed using the Clarity Western ECl Substrate (Bio-Rad) and signals were recorded using a Camera LAS 4000.

### Quantification of TNFα and TNFR_2_ expression

TNFα and TNFR_2_ protein expression was quantified as previously reported using Elisa R&D kits^26^.

### Quantification of cardiomyocyte area and tissue fibrosis

Frozen sections fixed in paraformaldehyde were labeled with Wheat Germ Agglutinin (WGA)-Alexa 647 (1/500 dilution, Molecular Probes). Tissue sections were analyzed with a Zeiss Axio Observer Z1 microscope using ImageJ software. A low *vs*. high threshold allowed quantification of cardiomyocyte area or tissue fibrosis, respectively, as previously reported^47^. Results were quantified from 7 mice/group (12 images/animal).

### Drugs

Isoproterenol (Sigma) and TNFα (R&D) were respectively used at 100 nM and 50 ng/mL, as previously reported^26^. Neutralizing anti-TNFR_1_ or anti-TNFR_2_ antibodies (R&D) were preincubated 1 hour at 37 °C at a final concentration of 2.5 µg/mL. All pharmacological inhibitors were preincubated for 10 min. Orai pharmacological inhibitors YM58483 (Tocris) and Synta66 (Servier) were added at 1 µM. PLA_2_ activating peptide (R&D) and the _c_PLA_2_ inhibitor, methyl arachidonylfluorophosphonate (MAFP) (Sigma), were respectively used at 20 µg/mL and 4 µg/mL. Lipoxygenase and cycloxygenase inhibitors, nordihydroguaiaretic acid (NDGA) and indomethacin (Sigma), were used at 1 µM. Montelukast (Sigma) was used at 2 µM. To explore ventricular myocyte resistance to oxidative stress, we applied 100 µM of H_2_O_2_ (Sigma). Semapimod (MedKoo) was used *in vitro* on CD11b/c cells at 10 µM.

### Statistical analysis

Quantitative data are represented using means and error bars indicating standard error of the mean (SEM) and were analyzed using XLStat 2014 (Addinsoft, New York, USA). Non-parametric Mann-Whitney U or Wilcoxon matched-paired tests were used for comparisons between two groups, when appropriate. Non-parametric Kruskal-Wallis test was used for comparisons between more than two groups. ANOVA for repeated measures followed by Dunn-Sidak post-hoc test was used to analyze differences in echocardiographic parameters over time. All values with p < 0.05 were considered significant.

## Supplementary information

Supplementary Information
