# Supplementary material for: Cardiac inflammatory CD11b/c cells exert a protective role in hypertrophied cardiomyocyte by promoting TNFR2- and Orai3- dependent signaling
Source: Sci Rep. 2019 Apr 15;9:6047. doi: 10.1038/s41598-019-42452-y (PMC6465256; doi:10.1038/s41598-019-42452-y)
Supplement: Supplementary file 1 — Supplementary Information [file 41598_2019_42452_MOESM1_ESM.pdf]

Cardiac inflammatory CD11b/c cells exert a protective role in hypertrophied cardiomyocyte by promoting $TNFR_2$- and Orai3- dependent signaling

Mathilde Keck*, Mathilde Flamant*, Nathalie Mougenot, Sophie Favier, Fabrice Atassi, Camille Barbier, Sophie Nadaud, Anne-Marie Lompré, Jean-Sébastien Hulot and Catherine Pavoine.

*Authors contributed equally to the work.

SUPPLEMENTARY METHODS

Immunofluorescence

Frozen cardiac sections (5µm) fixed in paraformaldehyde for 15 minutes at room temperature were stained immunohistochemically as previously reported[1-3] with the following antibodies: anti-CD11b/c (FITC-monoclonal, 1/50 dilution, Miltenyi), anti- TNFα (polyclonal goat anti-rat, 10µg/ml, R&D) revealed using Alexa Fluor 546 donkey anti-goat antibody (1/500 dilution, Molecular Probes, Invitrogen). Membranes were labeled with WGA-Alexa 647 (1/500 dilution, Molecular Probes).

Cells were fixed in 4% PFA, permeabilized and blocked with 0.5% Triton and 1% BSA. Staining were performed using antibodies described for tissue.

Tissue sections and cells were analyzed with a Zeiss Axio Observer Z1 microscope. Image analysis was performed using ImageJ and Photoshop CS5 (Adobe). Results are expressed as the number of positive cells per field and were quantified from 3-4 rats/group (10-14 images/animal).

Co-immunoprecipitation and western-blot

Isolated cardiomyocytes were lysed in 150 mM NaCl, 50 mM Tris pH=7.5, EDTA 5mM, 0.5% NP40, 1% triton, protease and phosphatase inhibitors cocktail (Sigma, France). Samples were then centrifuged at 1000 x *g* for 5 minutes to get rid of cell debris. Protein concentration was measured by the BCA Protein assay (Thermo Scientific, France). Ten µg of the anti-STIM1 (Alomone ACC-063) or a non-relevant antibody (histone 3, Abcam ab1791) were added to prewashed dynabeads protein G (Life technologies, France) at room temperature for 2 hours, followed by incubation with 250 µg of protein at 4°C overnight. The beads were washed 5 times with PBS-Tween 0.02% and the protein eluted with 30 µl 2X Laemmli sample loading buffer plus 30 µl glycine 50 mM pH 2.5 and heated to 70°C for 10 minutes. Thirty µl of immunoprecipitated samples or 80 µg diluted in 30 µL of input samples were run on a 4-12% Nu-PAGE gel (Life technologies, France), transferred to Hybond-C PVDF membrane according to the manufacturer protocol (Amersham Biosciences, GE Healthcare, France). Membrane was cut and each part was incubated with rabbit anti-STIM1 (1/500, Sigma, S6197), or rabbit anti-Orai3 (1/500, Prosci Inc 4117), rabbit anti-GAPDH (1/2500, Cell Signaling 2118) followed by anti-rabbit HRP (1/5000, Amersham Biosciences). Detection was performed using the ECL Western Blotting Substrate (Pierce) and signals were recorded using a Camera LAS 4000.

Quantitative RT-PCR

Table S4: **sequences of the primers used (5' to 3') for mouse in Figure S4.**

Nppa (mouse)	Fw 5'-TCGAGCAGATCGCAAAAGATC-3'
	Rv 5'-CTCACTAAACCACTCACTTAC-3'
Tnfα (mouse)	Fw 5'-CCTGTAGCCCACGTCGTAG-3'
	Rv 5'-GGGAGTAGACAAGGTACAACCC-3'

In vivo intramyocardial ultrasound-guided transthoracic siRNA delivery in mice

On-target plus Scramble and Orai3 siRNA (Dharmacon GE healthcare) were injected by ultrasound-guided transthoracic intramyocardial injection, as described in [4], at day 0 in control mice. Echocardiographic parameters were measured regularly as stated.

SUPPLEMENTARY REFERENCES

1 Defer, N. *et al.* The cannabinoid receptor type 2 promotes cardiac myocyte and fibroblast survival and protects against ischemia/reperfusion-induced cardiomyopathy. *FASEB journal : official publication of the Federation of American Societies for Experimental Biology* **23**, 2120-2130, https://doi:10.1096/fj.09-129478 (2009).

2 Wan, J., Benkdane, M., Alons, E., Lotersztajn, S. & Pavoine, C. M2 kupffer cells promote hepatocyte senescence: an IL-6-dependent protective mechanism against alcoholic liver disease. *Am J Pathol* **184**, 1763-1772, https://doi:10.1016/j.ajpath.2014.02.014 (2014).

3 Wan, J. *et al.* M2 Kupffer cells promote M1 Kupffer cell apoptosis: a protective mechanism against alcoholic and nonalcoholic fatty liver disease. *Hepatology* **59**, 130-142, https://doi:10.1002/hep.26607 (2014).

4 Kervadec, A. *et al.* Cardiovascular progenitor-derived extracellular vesicles recapitulate the beneficial effects of their parent cells in the treatment of chronic heart failure. *The Journal of heart and lung transplantation : the official publication of the International Society for Heart Transplantation* **35**, 795-807, https://doi:10.1016/j.healun.2016.01.013 (2016).

SUPPLEMENTARY FIGURES AND TABLES

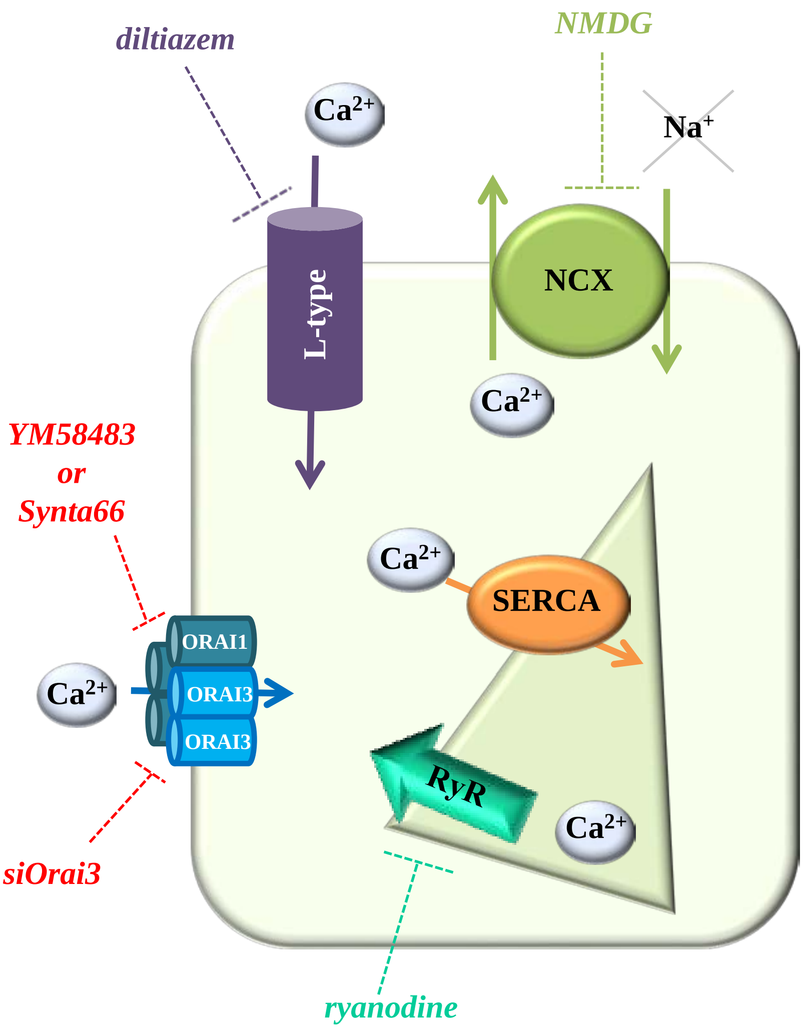

Figure S1: **Ca^{2+}-imaging protocol for the measurement of voltage- and store-independent Orai-dependent Ca^{2+} influx** in $Fura_2$-loaded cardiomyocytes.

	**Cardiomyocyte perimeters from normal rats**			
**Injection of siRNA**	**Scramble**	**Orai3**	**Scramble**	**Orai3**
***In vitro* 18 hrs post-treatment**	**No**		**Iso**	
**Mean cell area µm^2 ± SEM**	2540 ± 84	2496 ± 62	2617 ± 75	2677 ± 74
**Cell number**	113	118	111	103
***P* value**	*ns*		*ns*	

Table S1: **Neutralization of Orai3 in normal rats does not modify the cardiomyocyte area**. Cardiomyocytes were isolated from normal rats 3 days after intracardiac injection of Scramble or Orai3 siRNA. Cardiomyocyte area, in the absence of *in vitro* post-treatment, was not modify by Orai3 siRNA injection. *In vitro* isoproterenol treatment (iso 100nM 18 hrs) had a tendency to increase cardiomyocyte size, independently of injected siRNA. Typical experiments reproduced at least twice. Mean±SEM of cell area, n=103-118 cells, 3 rats/group, Mann Whitney U tests.

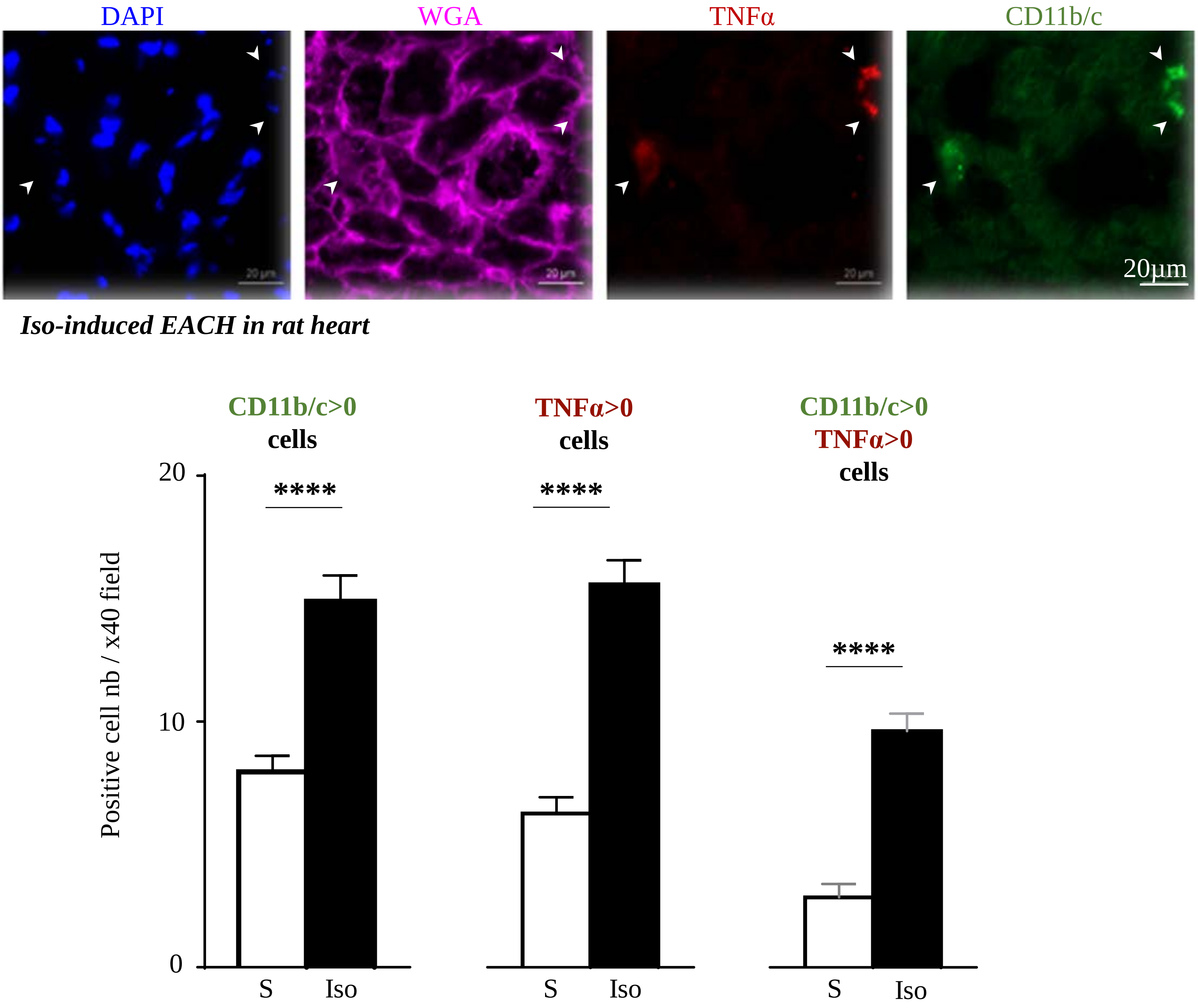

<u>Figure S2:</u> **Detection of an early cardiac inflammatory response associated with iso-induced EACH in rats.** Increased number of TNFα and CD11b/c positive cells detected by immunostaining in cardiac sections from iso-infused rats (Iso) (typical image), relative to saline-infused control rats (S), and quantification. Mean±SEM of fields, n=42 fields/group, 3 rats/group, 14 images/rat, Kruskal-Wallis followed by Dunn's post-hoc test, ****$p<0.0001$.

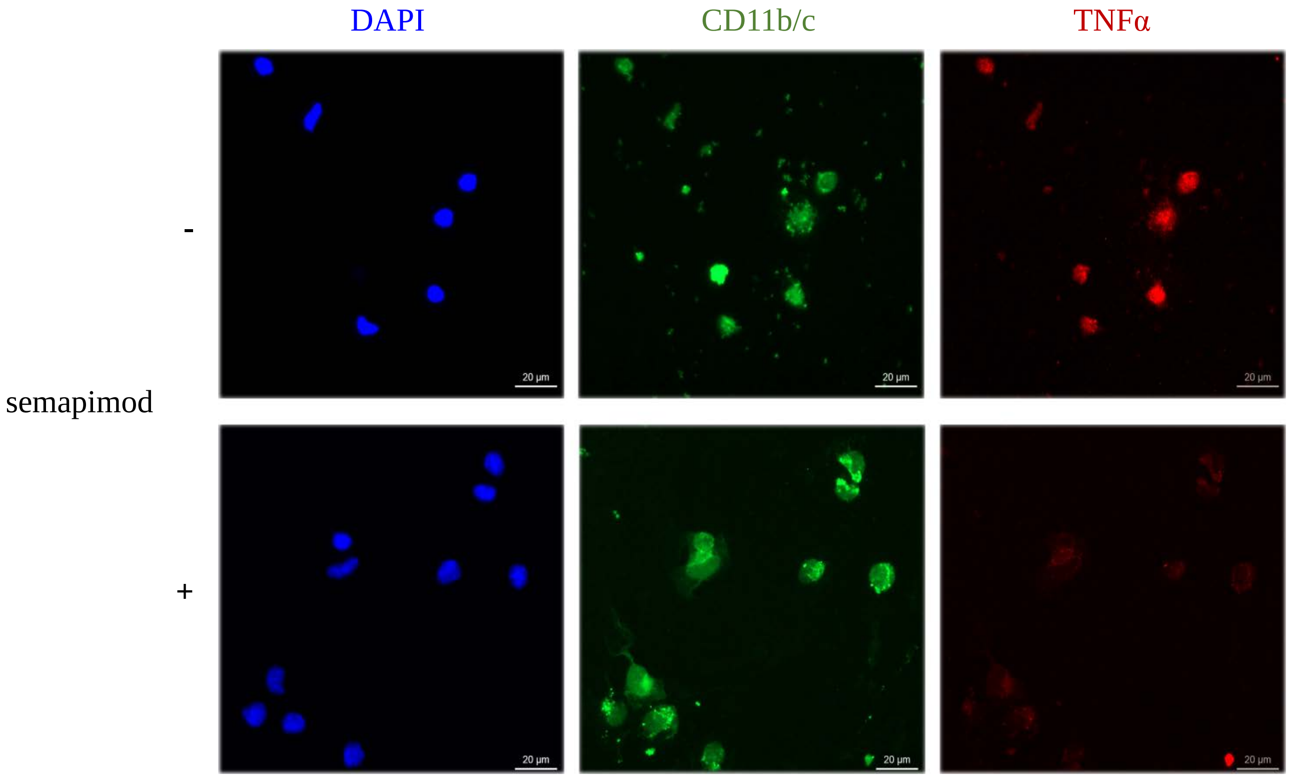

In vitro LPS-activated CD11b/c cells

Figure S3: **Anti-inflammatory impact of semapimod on *in vitro* LPS-activated CD11b/c cells.** TNFα immunostaining of CD11b/c cells isolated from a normal rat heart and pre-incubated with/without semapimod prior LPS application.

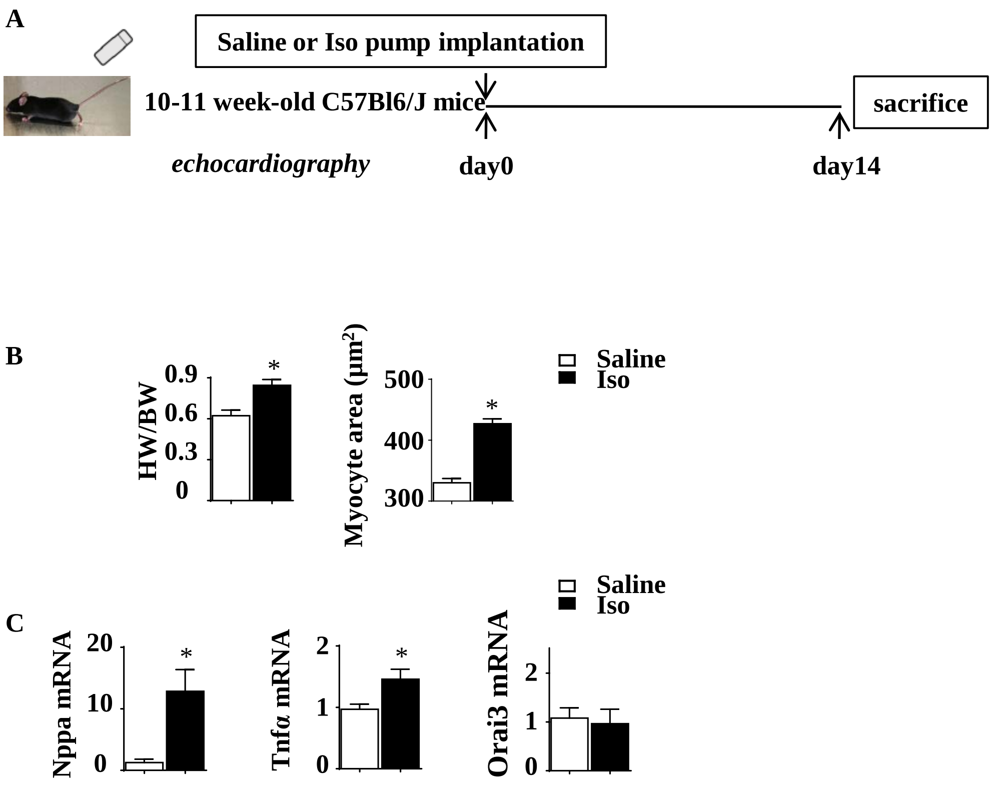

Figure S4: **Isoproterenol-induced EACH is associated with early cardiac inflammatory response.** (A) Schematic representation of the protocol where mice implanted with saline- or iso-pump for 14 days were subjected to echographic analyses. Iso-infusion induced (B) an increase in Heart Weight/Body Weight ratio (mean±SEM of mice, n=6 mice/group) and myocyte area (mean±SEM of cells, n=142-152 cells), (C) elevation of Nppa and Tnfα mRNA levels, but no modification of Orai3 mRNA expression (mean±SEM of mice, n=4-8 mice/group). Mann-Whitney U tests ,*$p<0.05$.

**Parameter**	**Saline at d14 (n=6)**	**Iso at d14 (n=6)**	***P* value**
**HR (bpm)**	585±7	630±8	p<0.05
**IVSd (mm)**	0.63±0.02	1±0.04	p<0.0001
**LVd (mm)**	3.85±0.12	3.88±0.18	*ns*
**PWd (mm)**	0.7±0.03	1.02±0.07	p<0.0001
**IVSs (mm)**	1.12±0.02	1.47±0.06	p<0.0001
**LVs (mm)**	2.22±0.03	2.02±0.21	*ns*
**PWs (mm)**	1.15±0.02	1.52±0.04	p<0.0001
**h/r**	0.35±0.004	0.53±0.02	p<0.0001
**EF (%)**	79.9±0.47	84.85±2.5	p<0.05
**FS (%)**	42.6±0.45	48.7±2.8	p<0.01

Table S2: **echocardiography parameters at day 14 in mice implanted with Saline or Iso pump at day 0.** Two-way ANOVA followed by Sidak’s post-hoc tests.

HR, heart rate; IVSd, end-diastolic interventricular septum thickness; LVd, end-diastolic left ventricular diameter; PWd, end-diastolic posterior wall thickness; IVSs, end-systolic interventricular septum thickness; LVs, end-systolic left ventricular diameter; PWs, end-systolic posterior wall thickness; h/r, diastolic wall thickness to radius ratio; EF, ejection fraction; FS, fractional shortening.

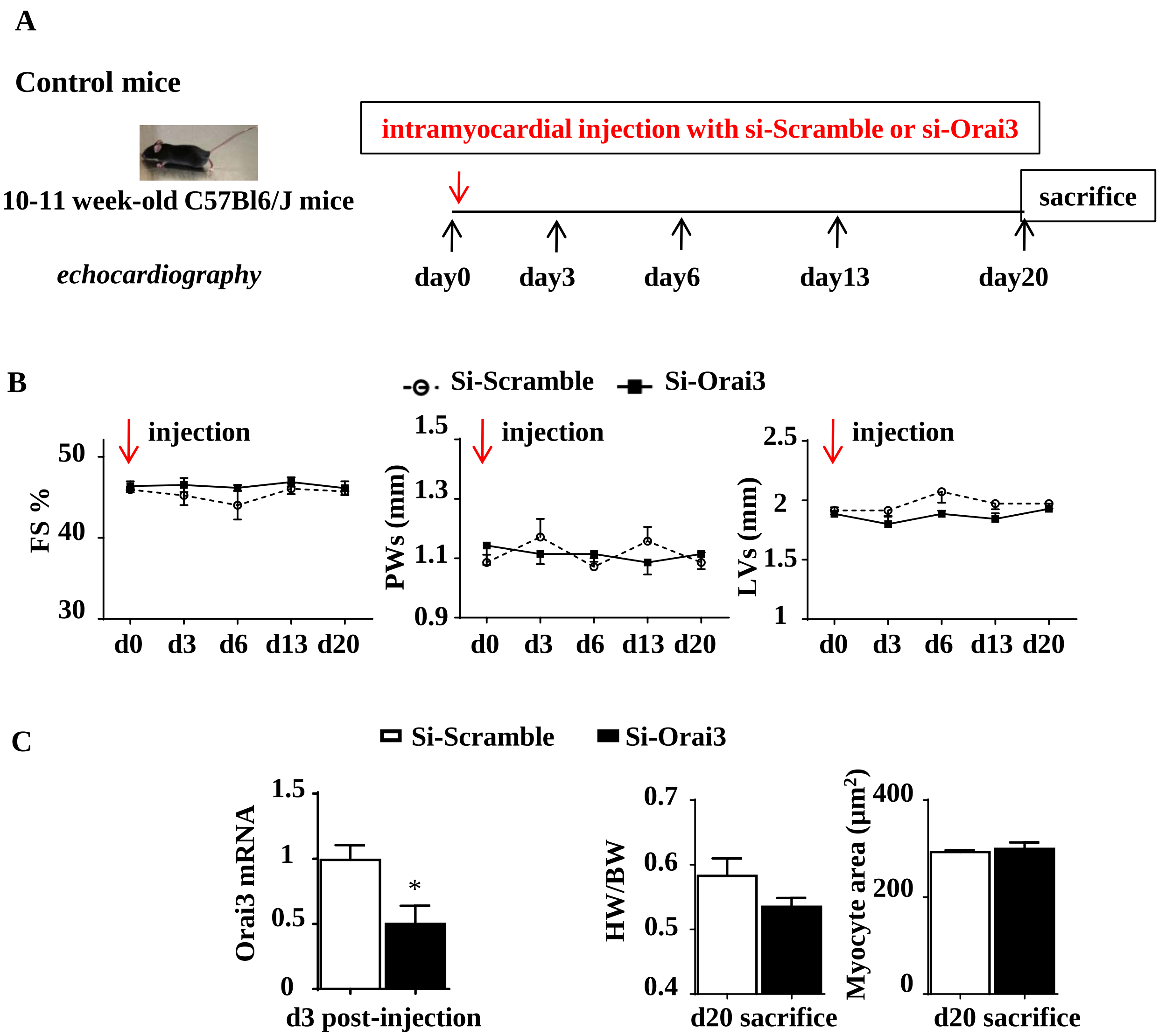

Figure S5: **Neutralization of Orai3 via intramyocardial injection of siRNA in control mice is without impact on remodeling and heart function.** (A) Schematic representation of the protocol where control mice were subjected to intramyocardial injection of Scramble or Orai3 siRNA. (B) Echocardiographic parameters (mean±SEM of mice, n=7 mice/group, two-way ANOVA followed by Sidak's post-hoc tests). (C) Efficient knockdown of Orai3 mRNA levels in cardiac homogenates at d3 following injection (mean±SEM of mice, n=5-10 mice/group, Mann-Whitney U test, *p<0.05). (C) SiOrai3 injection did not modify Heart Weight/Body Weight ratio and myocyte area (mean±SEM of mice, n=4-7 mice/group, Mann-Whitney U tests).

Control mice															
**Time of echocardiography**	**d0**			**d3**			**d6**			**d13**			**d20**		
**Injection at d0**	**Scr (n=7)**	**Orai3 (n=7)**	***p***	**Scr (n=7)**	**Orai3 (n=7)**	***p***	**Scr (n=7)**	**Orai3 (n=7)**	***p***	**Scr (n=7)**	**Orai3 (n=7)**	***p***	**Scr (n=7)**	**Orai3 (n=7)**	***p***
**HR (bpm)**	614± 5	616± 6	*ns*	639± 7	636± 8	*ns*	594± 10	617± 4	*ns*	632± 5	640± 4	*ns*	636± 10	642± 6	*ns*
**IVSd (mm)**	0.7± 0.02	0.6± 0.02	*ns*	0.7± 0.01	0.7± 0.01	*ns*	0.7± 0	0.7± 0.02	*ns*	0.7± 0.01	0.7± 0.01	*ns*	0.7± 0.02	0.7± 0.02	*ns*
**LVd (mm)**	3.5± 0.04	3.5± 0.09	*ns*	3.5± 0.07	3.3± 0.09	*ns*	3.6± 0.08	3.5± 0.05	*ns*	3.6± 0.07	3.5± 0.06	*ns*	3.6± 0.07	3.6± 0.06	*ns*
**PWd (mm)**	0.7± 0.03	0.7± 0.03	*ns*	0.7± 0.02	0.7± 0.02	*ns*	0.7± 0	0.6± 0.03	*ns*	0.7± 0.03	0.7± 0.03	*ns*	0.7± 0.03	0.7± 0.03	*ns*
**IVSs (mm)**	1.1± 0.03	1.1± 0.03	*ns*	1.1± 0.04	1.1± 0.02	*ns*	1.1± 0.03	1.1± 0.03	*ns*	1.2± 0.03	1.1± 0.01	*ns*	1.1± 0.02	1.1± 0.01	*ns*
**LVs (mm)**	1.9± 0.03	1.9± 0.05	*ns*	1.9± 0.04	1.8± 0.06	*ns*	2.1± 0.09	1.9± 0.02	*ns*	2± 0.04	1.8± 0.04	*ns*	2± 0.04	1.9± 0.04	*ns*
**PWs (mm)**	1.1± 0.02	1.1± 0.06	*ns*	1.2± 0.06	1.1± 0.03	*ns*	1.1± 0.03	1.1± 0.02	*ns*	1.2± 0.04	1.1± 0.04	*ns*	1.1± 0.03	1.1± 0.05	*ns*
**h/r**	0.38± 0.09	0.38± 0.08	*ns*	0.40± 0.09	0.40± 0.01	*ns*	0.38± 0.09	0.37± 0.01	*ns*	0.38± 0.08	0.38± 0.08	*ns*	0.36± 0.01	0.37± 0.01	*ns*
**EF (%)**	83± 0.27	83± 0.48	*ns*	82± 1.1	84± 0.74	*ns*	81± 1.8	83± 0.3	*ns*	83± 0.57	84± 0.32	*ns*	83± 0.37	83± 0.74	*ns*
**FS (%)**	45.9± 0.3	46.4± 0.5	*ns*	45.2± 1.1	46.5± 0.8	*ns*	44± 1.6	46.2± 0.3	*ns*	46.1± 0.6	46.9± 0.5	*ns*	45.7± 0.4	46.1± 0.79	*ns*

Table S3: **kinetics of echocardiography parameters in control mice after Scramble (Scr) or Orai3 siRNA intramyocardial injection.** Two-way ANOVA followed by Sidak's post-hoc tests.

HR, heart rate; IVSd, end-diastolic interventricular septum thickness; LVd, end-diastolic left ventricular diameter; PWd, end-diastolic posterior wall thickness; IVSs, end-systolic interventricular septum thickness; LVs, end-systolic left ventricular diameter; PWs, end-systolic posterior wall thickness; h/r, diastolic wall thickness to radius ratio; EF, ejection fraction; FS, fractional shortening.

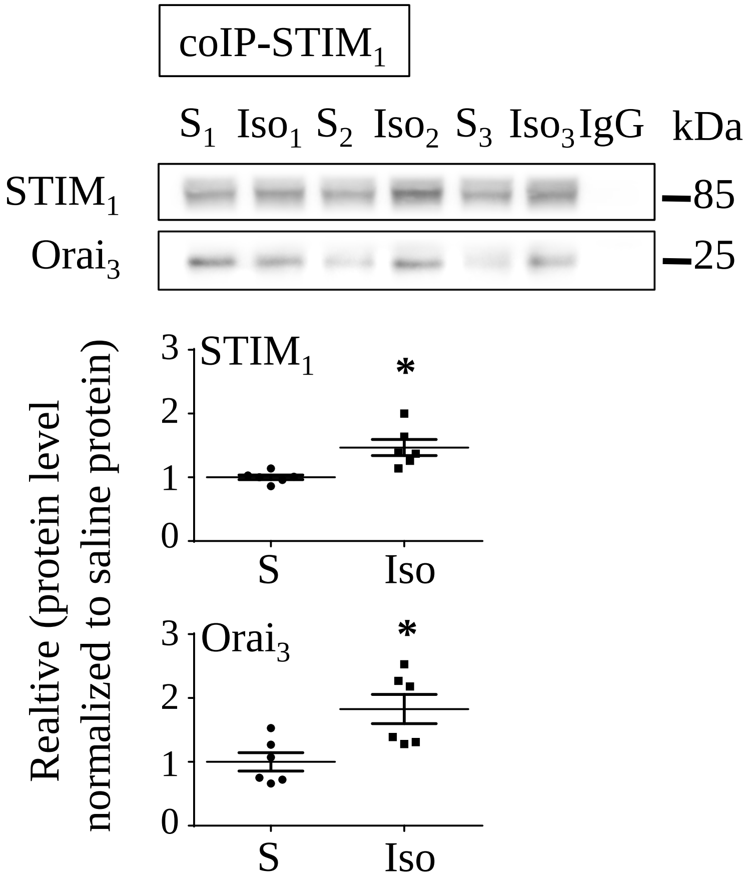

Figure S6: **Recruitment of Orai3 to STIM1 occurs in iso-induced hypertrophied cardiomyocytes.** STIM1 and Orai3 are present in the same macromolecular complex from cardiomyocytes isolated from rats implanted with saline(S)- or iso-pump(Iso). Co-immunoprecipitation of Orai3 with STIM1 in cardiomyocytes shows that a large recruitment of Orai3 occurs in cardiomyocytes derived from Iso rats. Each co-immunoprecipitation was repeated with cells isolated from three saline and three Iso rats. Iso values were normalized to mean saline for each blot. Statistical analysis was performed using a one-sample t test, testing if the mean of the Iso group differs from 1, $*p<0.05$. Full length blots were included in SI.

SUPPLEMENTARY INFORMATION

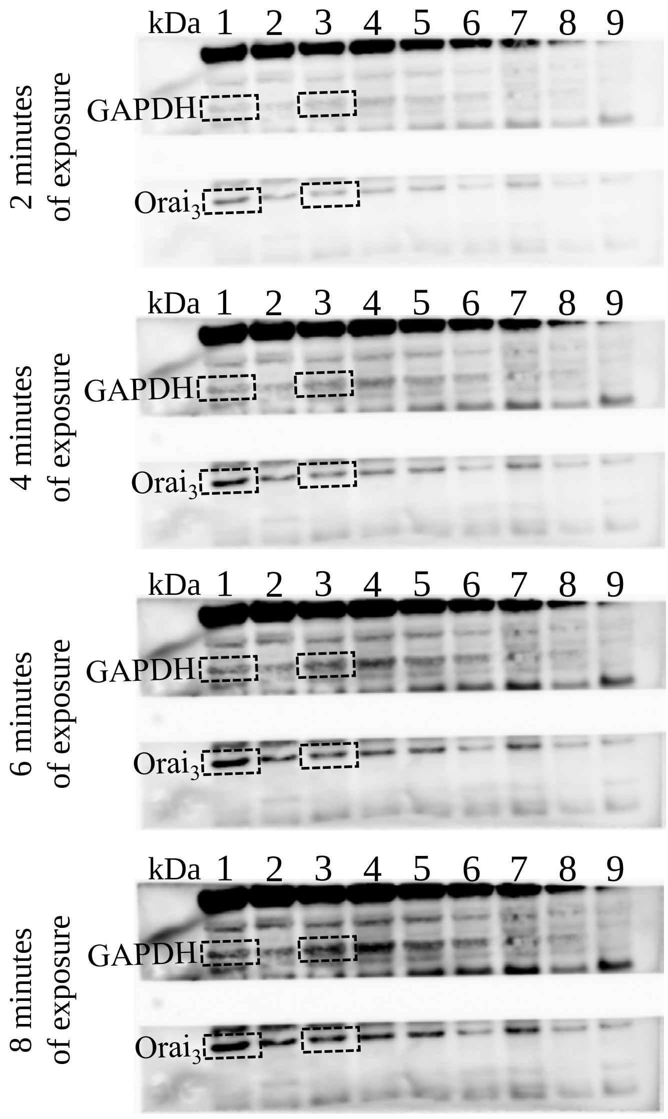

Supplementary information concerning Figure 2C: Blots of Figure 2C were obtained from lane 1 and lane 3 of the same Hybond-C PVDF membrane that was cut. Upper part of membrane was incubated with anti-GAPDH Ab, and lower part with anti-Orai3 Ab, followed by anti-rabbit HRP Ab. Detection of ECL signals was performed after reassembling of membrane parts and was recorded using a Camera LAS 4000. Times of exposures are indicated.

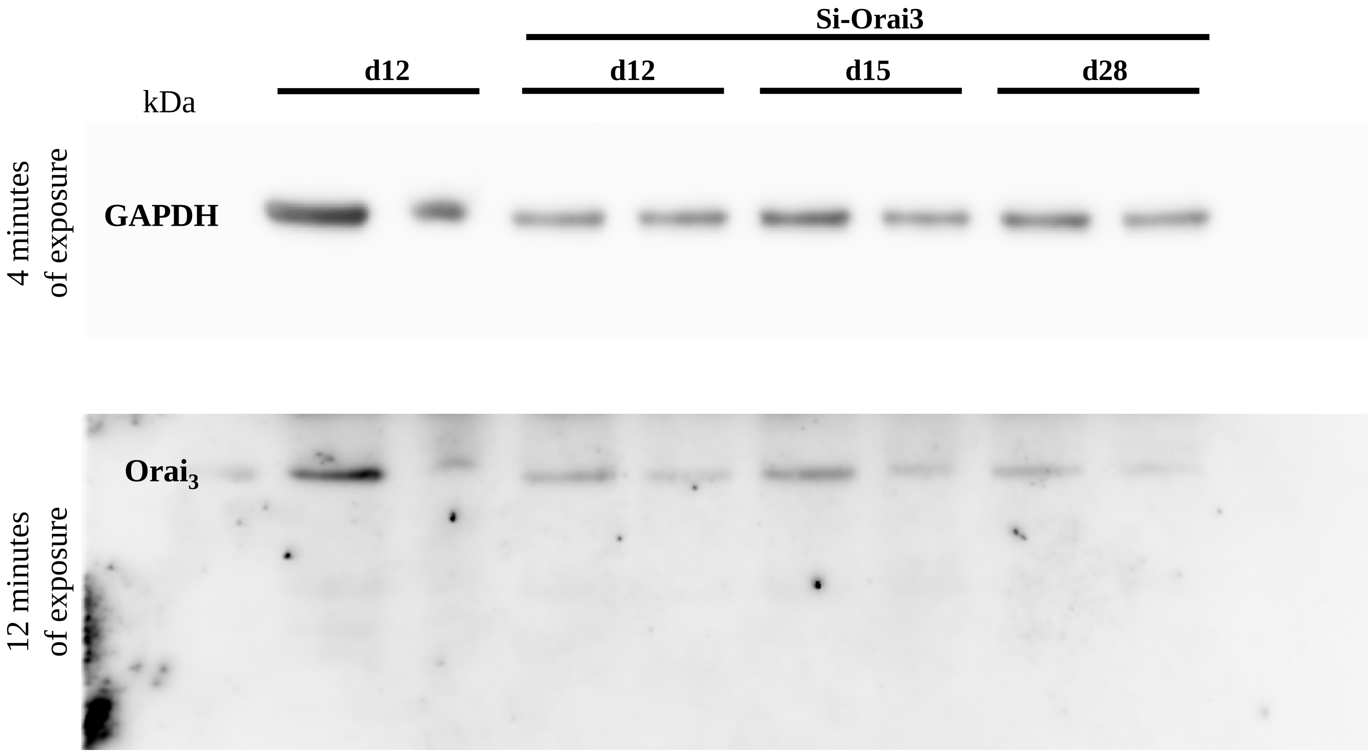

Supplementary information concerning Figure 8E: Blots of Figure 8E were obtained from the same Trans-Blot Turbo Mini-size nitrocellulose membrane (Bio-Rad) that was incubated with anti-GAPDH Ab or anti-Orai3 Ab, followed by anti-rabbit HRP Ab. Detection of ECL signals was performed using a Camera LAS 4000. Times of exposures are indicated.

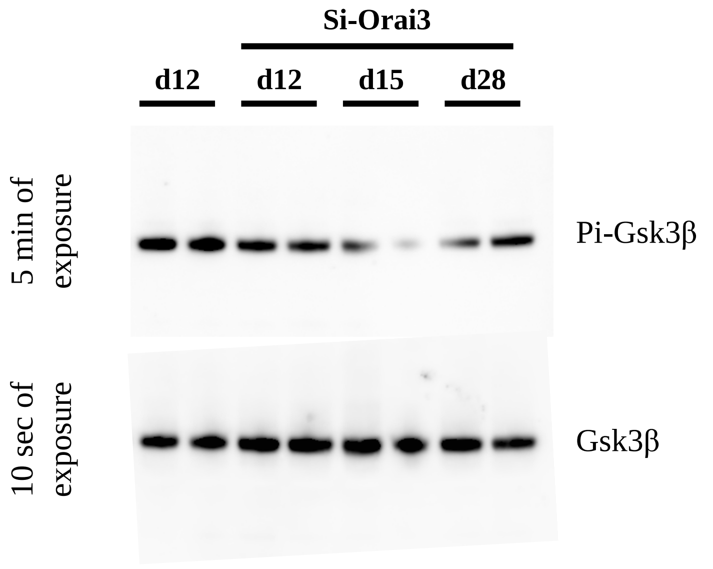

<u>Supplementary information concerning Figure 8H:</u> Blots of Figure 8H were obtained from the same Trans-Blot Turbo Mini-size nitrocellulose membrane (Bio-Rad) that was incubated with anti-Pi-GSk3β Ab, followed by anti-rabbit HRP Ab. Membrane was then immersed in stripping buffer (Restore PLUS Western Blot Stripping Buffer, Thermoscientific) for 7 minutes at room temperature, washed, blocked and incubated with anti-Gsk3β Ab, followed by anti-rabbit HRP Ab. Detection of ECL signals was performed using a Camera LAS 4000. Times of exposures are indicated.

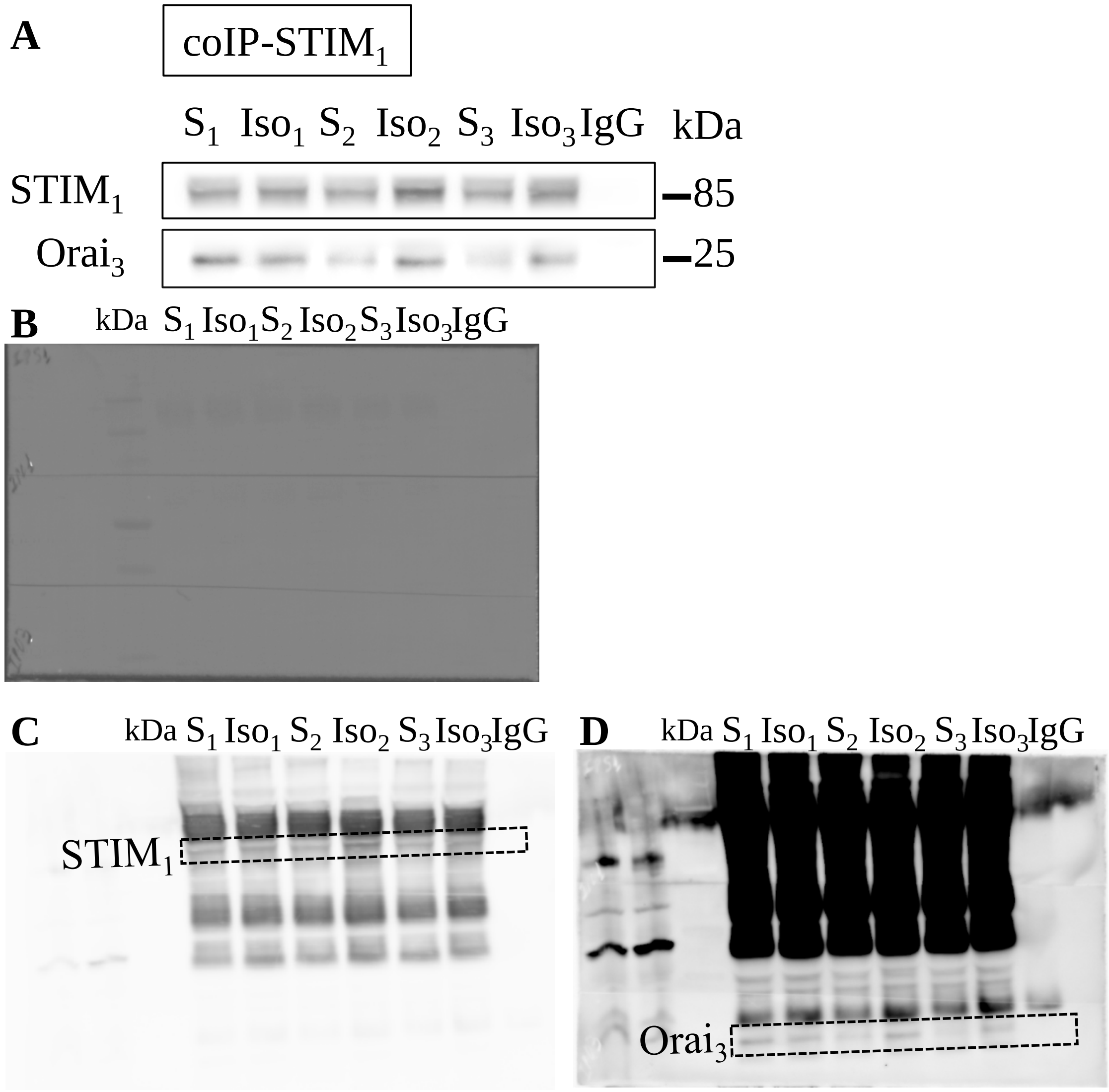

<u>Supplementary information concerning Figure S6:</u> (A) Blots of Figure S4 were obtained from the same Hybond-C PVDF membrane that was cut and each part was incubated with anti-STIM1 Ab, or anti-Orai3 Ab. Isolated cardiomyocytes from three saline and three Iso rats were lysed and centrifuged to get rid of cell debris. The anti-STIM1 Ab was added to prewashed dynabeads protein G at room temperature for 2 hours, followed by incubation with protein lysates from cardiomyocytes at 4°C overnight. The beads were washed and the proteins eluted. Immunoprecipitated samples were run on the same 4-12% Nu-PAGE gel, and then (B) transferred to the same Hybond-C PVDF membrane. (B-D) Membrane was cut and upper part was incubated with anti-STIM1 Ab, and lower part with anti-Orai3 Ab, followed by anti-rabbit HRP Ab. Detection of ECL signals was performed after reassembling of membrane parts and was recorded using a Camera LAS 4000. (C-D) Multiple exposures are represented.
